# The cost of host genetic resistance on body condition: Evidence from divergently selected sheep

**DOI:** 10.1111/eva.13442

**Published:** 2022-07-12

**Authors:** Frédéric Douhard, Andrea B. Doeschl‐Wilson, Alexander Corbishley, Adam D. Hayward, Didier Marcon, Jean‐Louis Weisbecker, Sophie Aguerre, Léa Bordes, Philippe Jacquiet, Tom N. McNeilly, Guillaume Sallé, Carole Moreno‐Romieux

**Affiliations:** ^1^ GenPhySE Université de Toulouse, INRAE, ENVT Castanet‐Tolosan France; ^2^ The Roslin Institute and Royal (Dick) School of Veterinary Studies University of Edinburgh Edinburgh UK; ^3^ Moredun Research Institute Penicuik UK; ^4^ P3R, INRAE Osmoy France; ^5^ UMR INRAE/ENVT 1225 IHAP, UMT Santé des Petits Ruminants Ecole Nationale Vétérinaire de Toulouse Toulouse cedex 03 France; ^6^ INRAE, UMR1282 ISP Université de Tours Nouzilly France

**Keywords:** host–parasite interaction, selection experiment, sheep, trade‐off

## Abstract

Trade‐offs between host resistance to parasites and host growth or reproduction can occur due to allocation of limited available resources between competing demands. To predict potential trade‐offs arising from genetic selection for host resistance, a better understanding of the associated nutritional costs is required. Here, we studied resistance costs by using sheep from lines divergently selected on their resistance to a common blood‐feeding gastro‐intestinal parasite (*Haemonchus contortus*). First, we assessed the effects of selection for high or low host resistance on condition traits (body weight, back fat, and muscle thickness) and infection traits (parasite fecal egg excretion and loss in blood haematocrit) at various life stages, in particular during the periparturient period when resource allocation to immunity may limit host resistance. Second, we analysed the condition–infection relationship to detect a possible trade‐off, in particular during the periparturient period. We experimentally infected young females in four stages over their first 2 years of life, including twice around parturition (at 1 year and at 2 years of age). Linear mixed‐model analyses revealed a large and consistent between‐line difference in infection traits during growth and outside of the periparturient period, whereas this difference was strongly attenuated during the periparturient period. Despite their different responses to infection, lines had similar body condition traits. Using covariance decomposition, we then found that the phenotypic relationship between infection and condition was dominated by direct infection costs arising from parasite development within the host. Accounting for these within‐individual effects, a cost of resistance on body weight was detected among ewes during their first reproduction. Although this cost and the reproductive constraint on resistance are unlikely to represent a major concern for animal breeding in nutrient‐rich environments, this study provides important new insights regarding the nutritional costs of parasite resistance at different lifestages and how these may affect response to selection.

## 1 INTRODUCTION

Trade‐offs among life‐history traits play a central role in evolutionary processes, but their underlying mechanisms need to be better understood to predict how they may constrain population responses to selection in diverse environments (Flatt & Heyland, 2011; Garland et al., 2022; Mauro & Ghalambor, 2020). Although resource allocation constraints due to limited resources are commonly assumed in life‐history evolution, the influence of such mechanisms on genetic changes is not straightforward when selection takes place in nutrient‐rich environments, as is usually the case in livestock populations artificially selected for defined traits (Douhard et al., 2021). Studies in livestock have stressed the negative side‐effects of selecting for high and fast growth rate or reproductive output on health traits (Rauw et al., 1998). However, it is less clear whether selection for health traits negatively affects growth or reproduction (Van Der Most et al., 2011), as would be expected under the resource allocation theory (Rauw, 2012). In theory, even in a nutrient‐rich environment, expressing traits such as host resistance to parasites during an infection could lead to a resource allocation trade‐off. Indeed, host resistance is nutritionally expensive to develop and maintain (Colditz, 2008; Lochmiller & Deerenberg, 2000), and nutritional limitations can occur during an infection due to host anorexia (Doeschl‐Wilson et al., 2009; Kyriazakis et al., 1998). Yet, how can we estimate these costs and their effect on health and productive responses to selection for host resistance?

Condition traits in the broad sense (e.g. body weight, condition score) reflect the level of host body resources and are often considered relevant indicators of host health, both in wild and domestic species (Liu, Smith, Karlsson, et al., 2005; Mavrot et al., 2015; Sánchez et al., 2018). Individuals in good condition are commonly expected to be more resistant to infections than individuals in poor condition that should be more susceptible (Beldomenico et al., 2008). The general relationship between condition traits and traits of infection severity should thus be negative. However, phenotypically, the direction and the strength of this ‘condition–infection relationship' are highly heterogeneous in the literature (Sánchez et al., 2018). Studies in quantitative genetics also tend to show that this relationship is inconsistent (Gold et al., 2019; Greer, 2008; Mucha et al., 2022). This high phenotypic and genetic heterogeneity among studies could well‐support diverse mechanisms, driving the condition–infection relationship in opposite directions. For instance, a high parasite burden may reduce foraging activity and lead to body reserves mobilization (resulting in a negative condition‐infection relationship), whereas hosts with high foraging activity may store reserves while ingesting more parasites (resulting in a positive condition–infection relationship; Sánchez et al., 2018). However, establishing the evolutionary importance of a particular mechanism such as resource allocation trade‐offs is out of reach when many factors affect the condition–infection relationship (e.g. resource availability, host physiological stage, parasite exposure, stage of infection) and cannot be well‐accounted for (Sandland & Minchella, 2003). Alternatively, selection experiments relying on artificial infection provide a means to focus on responses to selection in a specific host–parasite system, while controlling for the variation in the host genotype and environment (including the timing, duration, and virulence of infection) (Graham et al., 2011). Under those controlled conditions, the detection of a potential genetic trade‐off between resistance and condition would be facilitated but may still be hampered, in particular by the difficulty to differentiate infection costs.

**FIGURE 1:**
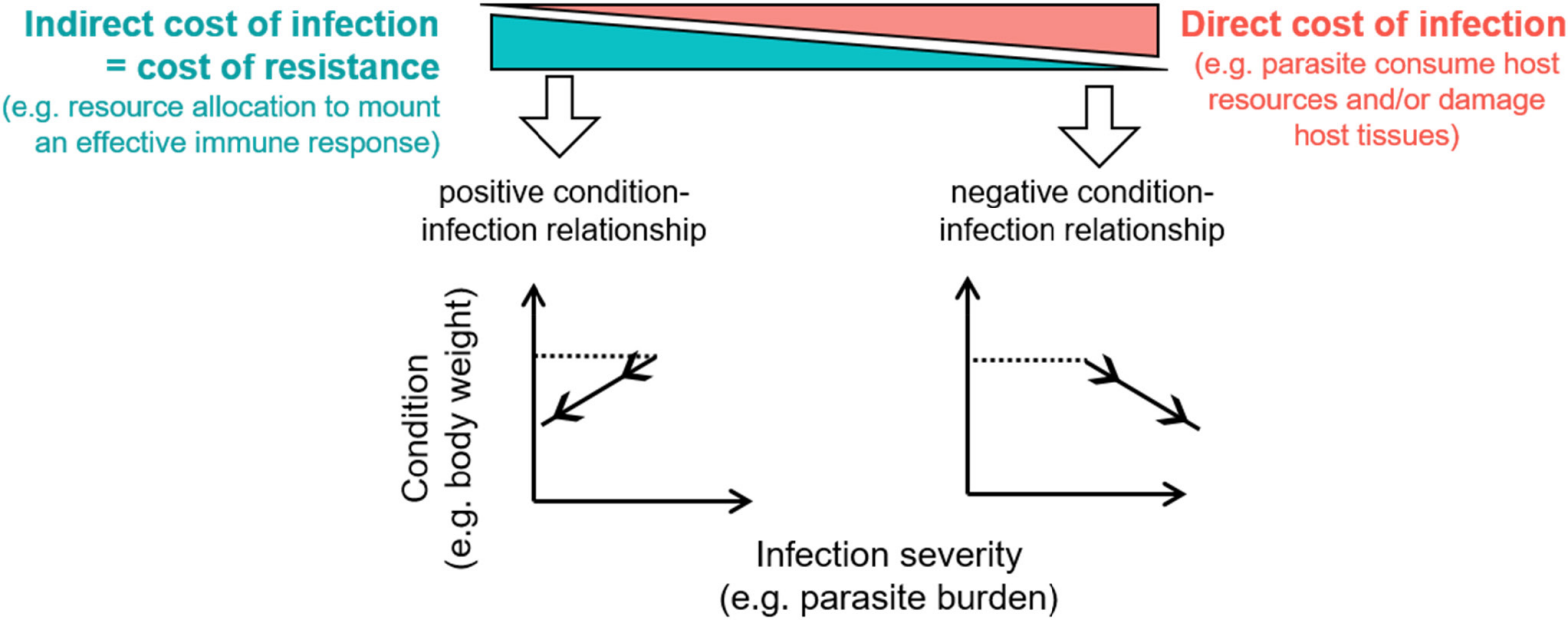
How the direction of the relationship between host condition and infection severity can relate to different costs of infection. Dashed lines indicate the start of the infection (transition of an individual from uninfected to infected) and continuous lines with arrows indicate the infection dynamic. For hosts with high resistance, indirect infection costs predominate and infection severity decreases (left hand side) whereas for hosts with low resistance, direct infection costs predominate and infection severity increases (right hand side)

Direct infection costs associated with parasite development have to be distinguished from indirect infection costs associated with the host immune response as only the latter captures the costs of resistance against parasites. Parasite resistance can involve costs on condition traits either through the allocation of nutrient intake towards immunity instead of being stored in tissues (Coop & Kyriazakis, 1999) or through a mobilisation of body reserves to fulfil the nutrient requirements of parasite‐specific immune responses (Kyriazakis et al., 1998). However, direct and indirect infection costs cannot be deduced from an overall decrease in host condition alone. Hypothetically, the relative importance of each type of cost may be inferred from the simultaneous change in parasite burden (Cressler et al., 2014), and thus from the direction of the condition–infection relationship (Figure 1). As parasite burdens increase, the exploitation of host resources (i.e. direct infection costs) by the parasites is expected to increase. In the instance where the host only allocates a small amount of resource towards immunity (as can typically happen during reproduction; French et al., 2007), the costs of resistance (i.e. indirect infection costs) are small, relative to the direct costs. In the instance where the host mounts an effective immune response (i.e. demonstrates disease resistance), then the indirect costs of infection predominate relative to the direct costs. Of course, one must also consider the scenario where the host mounts an ineffective immune response, in which case both the direct and indirect costs of infection are high. The absence of an infection–condition relationship would not allow one to determine whether direct and indirect costs both occur to the same extent or whether none of those costs occur at all. However, the condition–infection relationship may provide insight into the relative importance of host resistance costs in extreme situations where direct or indirect costs predominate and can be compared.

Divergent selection experiments provide a tool to generate such extreme situations (Garland, 2003) so that previous hypotheses can be tested. In this study, we used females from domestic sheep lines divergently selected on their resistance to a common blood‐feeding gastrointestinal parasite (*Haemonchus contortus*). Gastro‐intestinal parasitism in sheep is a well‐studied host–parasite system that is both ecologically relevant and that has strong practical implications in livestock (Hayward, 2013). Host resistance to gastro‐intestinal parasites, typically approximated by fecal egg count (FEC), exhibits genetic variation in diverse sheep populations, which provides opportunities to reduce gastro‐intestinal parasite burden and its impact on livestock production by selecting animals with greater genetic resistance (Bisset & Morris, 1996; Stear et al., 2001). In addition, resource allocation constraints have long been thought to be prominent in this host–parasite system, in particular in reproducing females around their lambing where an insufficient allocation of metabolizable protein may limit the expression of immunity against gastro‐intestinal parasites (i.e. the so‐called periparturient relaxation of immunity [PPRI]; Barger, 1993; Houdijk et al., 2001). Even if the immunosuppressive effects of reproduction are well‐acknowledged, their consequences for selection for host resistance remain poorly understood. In particular, if resource allocation constraints do occur, we do not know to which extent and how they might be circumvented through selection on host resistance. Population studies in sheep have shown strong positive genetic correlations between lamb resistance and ewe resistance (Bishop & Stear, 2001; Brown & Fogarty, 2017; Goldberg et al., 2012; Notter et al., 2018; Pollott et al., 2004). Sheep lines that have been selected for high resistance also exhibit consistent responses to infection across life stages (Morris et al., 1997; Woolaston et al., 1990), although ewes from those resistant lines still undergo a PPRI (Morris et al., 1998; Woolaston, 1992). Yet, the question of a potential cost of ewe resistance on condition traits, in particular during the periparturient period, remains mostly unexplored so far. Accordingly, the objectives of this study were twofold: (i) to assess the effects of selection for high or low host resistance on several ewe condition traits (body weight, back fat, and muscle thickness) and infection traits (FEC and loss in haematocrit) over growth and reproduction, in particular during the peripartum period when dietary protein may limit the expression of host resistance, and, (ii) to analyse the condition–infection relationship and detect a possible trade‐off, in particular during the periparturient period. For this, we artificially infected genetically resistant (R) and susceptible (S) ewes in four stages over the first 2 years of life, including twice around lambing. At a same level of reproductive expenditure, selection for costly resistance should reduce the degree of PPRI in the R line compared to the S line, but if resistance costs are relatively high, then negative effects should be observed among the various condition traits that we measured in R but not S ewes, consistent with the notion of a trade‐off. Moreover, given the nutritional basis of the PPRI (Houdijk et al., 2001), the putative trade‐off may only be revealed under protein restriction. Hence, we also tested the effects of varying the level of dietary protein when infecting ewes during the periparturient period. Finally, to better understand how trade‐offs and selection may shape the condition–infection relationship (objective ii), we linked the direct and indirect infection costs occurring at the within‐individual level (Figure 1) to selection responses observed at the among‐individual level. For this, a statistical framework that allows to detect trade‐offs at these different levels has been applied (Careau & Wilson, 2017) and is described below.

## 2 MATERIALS AND METHODS

The experimental procedures described hereafter were approved by the French Ministry for Higher Education and Research and the Centre Val de Loire ethics committee under the experimental approval D18‐174‐01.

### 2.1 Animals

#### 2.1.1 Selection experiment

The divergent selection on lamb resistance to *H. contortus* took place indoors in an experimental sheep farm (INRAE La Sapinière, Osmoy, France). It is described in detail by Sallé et al. (2021). At each generation, we collected FEC as a measure of parasite resistance following a protocol of artificial infection with third‐stage larvae (L3) of *H. contortus* from the strain ‘Humeau’ (Lacroux et al., 2006). The protocol was made up of two successive infections of 10,000 L3/sheep and lasted 11 weeks: first, naïve lambs were infected to stimulate a primary immune response; 4 weeks later they were treated (0.2 mg/kg of live weight of ivermectin; Oramec, Boerhinger Ingelheim, Lyon, France); after 2 weeks of recovery they were re‐infected to simulate a secondary immune response and finally treated 5 weeks later. At the end of first and second infection, FEC was recorded just before treatment. Estimated breeding values (EBVs) of these two FECs were computed using a model including fixed effects (lamb age, group pen, body weight, litter size, and sex) and an individual random effect estimated from the pedigree relationship matrix. The two EBVs per animal were then combined and used to select in each line 2–5% of males with most extreme EBVs as fathers for the next generation (Sallé et al., 2021). Mating was planned to limit inbreeding.

#### 2.1.2 Study animals

We used 91 female sheep (51 R and 40 S) from the second generation of selection (G_2_). At G_2_, the divergence in FEC between R and S sheep reached 1.9 phenotypic SD (σ_p_) and 3.8 genetic SD (σ_g_) calculated from the initial population (G_0_) (Sallé et al., 2021). The 91 females were produced from seven G_1_ sires (3 R and 4 S) mated with 37 G_0_ rams (22 R and 15 S) and 32 G_1_ rams (14 R and 18 S). Lambs were born between August 29th and October 10th 2017. They were approximately 4 months old (*SD* = 8.3 days) at the beginning of the experiment, were not previously exposed to *H. contortus*, and were housed indoors during the whole experiment to prevent natural infection by gastro‐intestinal nematodes. Lambs from both lines were reared by their mother until 2 months of age and were then kept together in four pens and managed identically.

### 2.2 Experimental design

**FIGURE 2:**
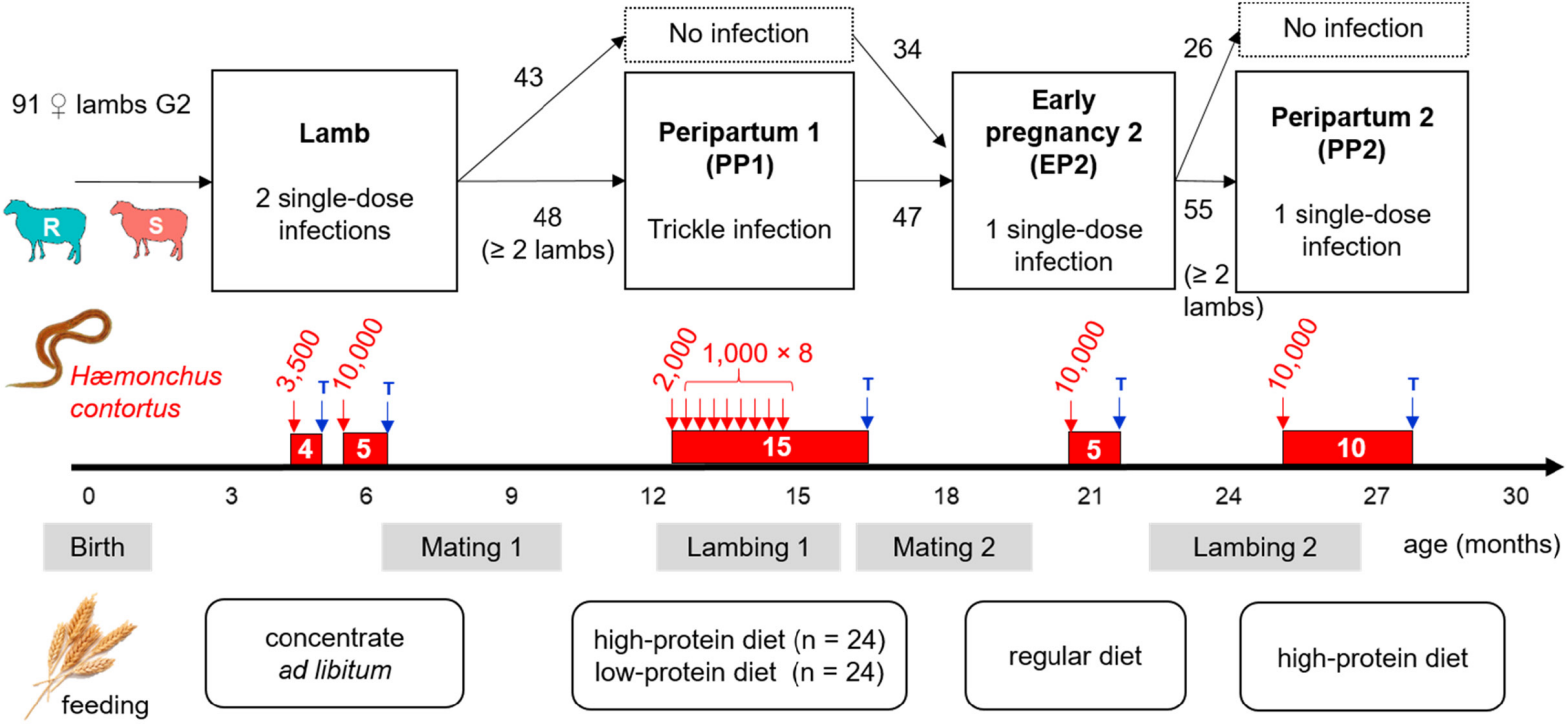
Overview of the experimental design based on 91 female lambs from the second generation of lines selected for parasite resistance (R) or susceptibility (S). Red areas indicate infection periods (in white: Duration in weeks; in red: Number of third‐stage larvae of *Haemonchus contortus* orally given per sheep). Each time, infections were ended by antihelmintic treatment (T)

The experimental design included artificial infections in four stages over the first 2 years of life (Figure 2). At 4 months of age, all 91 female lambs went through the infection protocol used to phenotype parasite resistance (as described in section 2.1.1), except that the first dose was of 3500 L3/sheep to limit the potential negative consequences of infection on fertility at first mating (at 8–9 months of age). During the infection, lambs were fed *ad libitum* with a protein‐rich concentrate and straw (see diet composition in Appendix S1). A second phase of infection of 15 weeks started about 4 weeks before lambing (peripartum) and was based on a trickle infection (about 1000–2000 L3/ewe/week during the first 9 weeks). During this phase (PP1), a 2 × 2 factorial experiment was set up to test the effect of a dietary protein restriction on parasite resistance. Half of the R and S ewes were fed at 70% of their calculated protein requirements, whereas the other half of ewes were fed at 120% of their dietary protein requirements (the composition and daily amounts of feed concentrate required to provide 70% and 120% of the calculated protein requirements were determined according to the INRAE feeding system (INRA, 2018); Appendix S1). Feed concentrates were isoenergetic, and daily amounts were distributed individually with automatic feeders, with straw available *ad libitum*. To limit individual variation in parasite resistance that could arise from variation in litter size, we only used ewes bearing multiple lambs in this phase of infection (24 R and 24 S). Moreover, litter size was reduced to a maximum of two lambs per ewe during lactation. Extra lambs were moved to artificial rearing. After weaning and second mating, we proceeded to a third infection phase during early pregnancy of the 2‐year old ewes (EP2). This included the 81 females that remained from the 91 initially involved. At the end of this pregnancy, we set up a last phase of infection during the periparturient period (PP2). Contrarily to PP1, PP2 was based on a single‐dose infection of 10,000 *H. contortus* L3 to assess if responses to infections during the periparturient period might depend on the mode of infection. As for PP1, only ewes with at least two lambs born were involved (*n* = 55). As we did not detect an effect of protein restriction on FEC during PP1, we did not apply the feeding treatment during PP2 and rather preferred to increase the statistical power of our design to focus on a potential line effect on FEC around lambing. We thus fed ewes with the same high‐protein diet of PP1 (120% of calculated protein requirements) to ensure that insufficient protein was not limiting parasite resistance during this phase.

### 2.3 Measurements

#### 2.3.1 Infection traits

Fecal egg count and the loss in blood haematocrit during the infection were considered proxies of worm burden. Low FEC is usually associated with high host resistance as it approximates the overall animal capacity to prevent L3 establishment in the abomasum and their development into fertile adults. Accordingly, it was chosen as the criterion subject to divergent selection in this experiment (cf. section 2.1.1). Blood haematocrit (HE) may be less directly related to host resistance as anaemia is not specific to infection, and host change in HE reflects both the damage caused by the blood‐feeding activity of the parasite burden and host tolerance to this damage.

Both FEC and HE were measured repeatedly over the four phases of infection. Within 48 h following the collection of faecal samples, FEC was measured using the modified McMaster technique (Raynaud et al., 1970). On each day of faecal sampling, blood was also taken from the jugular vein to measure HE by the microhaematocrit centrifugation technique. Each animal was sampled the day of infestation (day 0) to check that FEC was null and obtain a baseline level of blood haematocrit (HE_0_) from which the following change at time *t* of infection relative to the baseline was defined (i.e. ΔHE_*t*_ = HE_0_–HE_*t*_). Hence, as for FEC, ΔHE_*t*_ should be positively related to worm burden. To characterize the changes in FEC and ΔHE over the course of infections, we sampled animals weekly from week 3 when egg excretion should start (day 21) until week 15 (PP1), week 5 (EP2), or week 10, (PP2). During the lamb phase, sampling was more frequent (every 3 or 4 days from day 21 to day 35) to describe in finer details temporal changes in FEC and ΔHE in 42 lambs with the most extreme EBVs among the total sample of 91. The 49 other lambs were sampled four times (start and end of each infection) as normally carried out in the phenotyping protocol (section 2.1.1).

#### 2.3.2 Condition traits

We measured body weight (BW) in all infected animals every day of faecal sampling and during additional days (Table 1). On 29 of the 34 time points, animals were weighed and we also performed dorsal ultrasound scans to measure back fat (BFT) and muscle thickness (MT) on the left and the right at the 12th–13rd lumbar vertebra (Easi‐Scan™, IMV imaging). Individual values of BFT and MT were defined as the average of the measures performed on the left and right sides of the animals.

**TABLE 1:** Number of the different individual measurements analysed during the experiments (with corresponding number of time points). ‘Lamb’: Single‐dose infection at 4–5 months of age; ‘PP1’ or ‘PP2’: Peripartum infection at 1 or at 2 years of age, respectively; ‘EP2’: Single‐dose infection during early pregnancy at 2 years of age

Measurements	Stage of infection			
	Lamb	PP1	EP2	PP2
Fecal egg count (per gram of feces)^a^	259 (5)^b^	515 (11)	243 (3)	314 (6)
Loss in blood haematocrit (%)^a^	299 (6)^b^	578 (13)	243 (3)	314 (6)
Body weight (kg)	637 (7)	767 (16)	324 (4)	372 (7)
Backfat thickness (mm)	343 (6)^b^	725 (15)	243 (3)	261 (5)
Muscle thickness (mm)	343 (6)^b^	725 (15)	243 (3)	261 (5)
Concentrate intake (kg/day)^c^	Yes	Yes	No	Yes
Milk composition (% fat, % protein, somatic cell count)	No	48 (1)	No	55 (1)
Lamb traits from birth to 1 month^d^	No	Yes	No	Yes

^a^ Excluding control measures at day 0 of infection.

^b^ Only on 42 animals among the total sample of 91, except at day 0 and at the last day of infection.

^c^ Automatically recorded daily. *Ad libitum* access during the lamb phase; restricted feeding in other phases.

^d^ Litter size, lamb body weight at birth and at weaning.

During the lamb phase, voluntary feed intake (kg/day) was recorded individually as feed concentrate was distributed *ad libitum* by automatic feeders. The same devices were used during PP1 and PP2 to provide the limited amounts of concentrate. From the automatic records, we checked that the predefined amounts of concentrate were effectively consumed. During PP1 and PP2, several lamb traits were measured: litter size and BW at birth, as well as BW gain of lambs from birth to 1 month. At about 1 month of lactation, we also took milk samples of ewes and sent them at the Interprofessional Milk Analysis Laboratory (Agrolab's Aurillac, France) to analyse milk composition using Milko‐Scan™ FT6000 (Foss, Nanterre, France) (Table 1).

### 2.4 Statistical analyses

#### 2.4.1 Differences between selection lines in infection traits and condition traits (univariate models)

To assess line differences (first objective of this study), we analysed separately the changes in the two infection traits (FEC and ΔHE) and in three condition traits (BW, BFT, and MT) during each phase of infection. During PP1 and PP2, data observed during pregnancy and lactation were analysed separately. To assess the effect of line on trait changes (R vs. S) while accounting for the non‐independence of the data, we implemented linear mixed models following the standard protocol described by Zuur et al. (2009) and using the ‘nlme’ package (Pinheiro et al., 2019) in R v3.5.0 (R Core Team, 2018). Infection and condition traits used as dependent variables were normally distributed, except FEC that was transformed to account for skewness in its distribution. For each phase of infection, two transformations were tested (i.e. $\log\left(\mathrm{FEC}+1\right)$ or $\sqrt{\mathrm{FEC}}$), and we selected the best transformation based on residual distribution assessment. Log‐transformation was chosen for the lamb infection and pregnancy of PP1 and of PP2, whereas square‐root transformation was preferred for lactation of PP1 and of PP2. Although generalized linear models are often more suitable than linear models for overdispersed parasite data, they are also more challenging to fit, especially for multivariate models with repeated measurements. Here, we preferred linear modelling to rely on a consistent framework where we first focus on the different fixed effects for each trait, before addressing covariance between traits (section 2.4.2 on multivariate modelling).

For each phenotypic observation of trait *y* from individual *i* observed at time *t*, sheep identity was fitted as a random effect (ind_*i*_) to account for the repeated measurements over time of infection. This effect and the random residual effect ($e_{\mathrm{it}}$) were assumed to follow a normal distribution (N(0, Ω_ind_) and N(0, Ω_*e*_), respectively), where the variance Ω_ind_ and Ω_*e*_ were estimated as *V*_ind_ and *V*_*e*_, respectively:

(1) $$y_{\mathrm{it}}=\beta_{0}+\sum_{s=1}^{n}\beta_{s}x_{\mathrm{sit}}+{\mathrm{ind}}_{i}+e_{\mathrm{it}},$$

with *β*_0_ the general intercept and *β*_*s*_ the *s*th fixed effect of predictor *x*_*sit*_. The *n* potential fixed‐effects predictors *x* that were considered for each infection phase are described in Table 2. Considering all those potential fixed effects, we first tested several error variance structures for Ω_*e*_, including heterogeneous variances (‘VarIdent') with time of infection or with line (i.e. line‐ or time‐specific *V*_*e*_), combined with several temporal correlation structures (first‐order auto‐regressive, linear, exponential) to account for the potential dependence between residuals *e*_*kt*_ and *e*_kt'_ corresponding to different times *t* and *t*'. The choice of variance structure was based on Akaike information criterion corrected for small sample size (AICc), where AICc was calculated in models containing all fixed effects and interactions under consideration. Once the variance structure Ω_*e*_ has been chosen, we looked for a more optimal fixed structure by implementing model selection (Burnham & Anderson, 2002) with the R package ‘MuMin’ (Barton, 2019). In all candidate models, the potential fixed effects (Table 2) were subject to selection, except ‘Line’, day of infection (‘Day’; considered categorical), and their interaction (‘Day × Line’) that were systematically included as the changes in the various traits for the two lines were of primary interest here. Once the best model has been selected, least‐square means were computed and between‐line differences were assessed using pairwise comparisons in the package ‘emmeans' (Lenth, 2022).

**TABLE 2:** List of fixed effects tested and included (•) or tested but not included (o) in the univariate linear mixed models selected to assess differences between lines during each stage of infection

Factor or variable included as fixed effect	Levels of factor^a^	Stage of infection					
		Lamb	PP1		EP2	PP2	
			P	L		P	L
Line; day; (day × line)^b^	R; S	•••••	•••••	•••••	•••••	•••••	•••••
Diet; (diet × line); (day × line × diet)	Low protein; High protein		o••••	o••oo			
Pen	A, B, C, D	oo••o					
Age	Linear continuous	ooooo					
Days from lambing 1	Linear continuous		oo•oo	ooooo			
Number of lamb born at 1 year old (‘NLB1’)	2; >2		oo•oo	ooooo	•oooo	ooooo	ooooo
Number of lamb suckled at 1 year old (‘NLS1’)	1; 2			ooo•o	ooooo	ooooo	ooooo
Days from lambing 2	Linear continuous					ooooo	ooooo
Number of lamb born at 2 years old (‘NLB2’)	2; >2				ooooo	ooooo	ooo•o
Number of lamb suckled at 2 years old (‘NLS2’)	1; 2						oo•o•
Treatment during PP1	Infected and high‐protein; Infected and low‐protein; Non‐infected				o••oo	oooo	o•ooo

*Note*: ‘Lamb’: Single‐dose infection at 4–5 months of age; ‘PP1’ or ‘PP2’: Peripartum infection at 1 or at 2 years of age, respectively; late pregnancy (‘P’) and lactation (‘L’) analysed separately; ‘EP2’: Single‐dose infection during early pregnancy at 2 years of age. At each stage of infection, the five points indicate the inclusion or not of the first factor indicated in each row in the following order of variables: (1) fecal egg count (FEC), (2) loss in blood haematocrit (ΔHE), (3) body weight (BW), (4) backfat thickness (BFT), and (5) muscle thickness (MT) (e.g. ‘diet' during pregnancy in PP1 [o••••] is included in the selected model for all traits expect FEC).

^a^ Correspond to the first factor indicated in each row.

^b^ Factors systematically included in the selected model for the five traits.

#### 2.4.2 Statistical framework for detecting trade‐offs (multivariate models)

**FIGURE 3:**
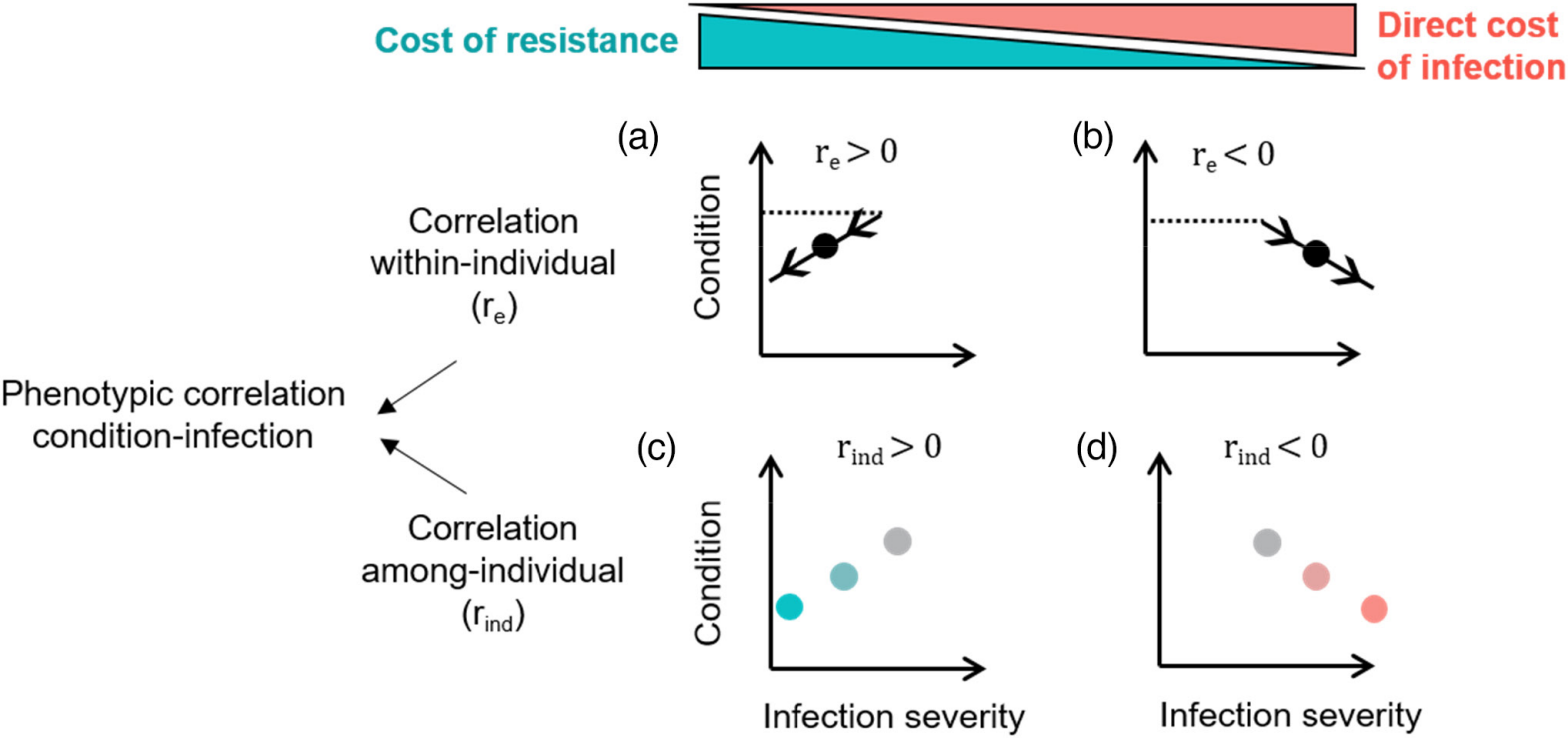
Decomposition of the condition‐infection relationship at the within‐individual level (a and b) and at the among‐individual level (c and d). This extends the link between the condition‐infection relationship and the different infection costs (indirect or direct), as presented in Figure 1. The direction of the relationship is driven by the within‐individual correlation (*r*_*e*_; a and b) and the among‐individual correlation (*r*_ind_; c and d). Within‐individual lines indicate the start of the infection (dashed; transition of an individual from uninfected to infected), and the infection severity either decreases for hosts with high resistance (a) or increases for hosts with low resistance (b). Dots represent individual averages during the infection. If the mechanisms acting within‐individual were strongly determining responses to selection observed among individuals, then selection for high host resistance should produce a positive relationship (c; where the increasing shade of blue indicates higher genetic resistance characterized by high indirect [resistance] costs of infection). Conversely, selection for low host resistance should produce a negative relationship (d; where the increasing shade of red indicates lower genetic resistance characterized by high direct costs of infection)

To analyse how trade‐offs and selection may shape the condition–infection relationship (second objective of the study), it is key to disentangle this relationship at the within‐ and among‐individual levels. Indeed, if direct or indirect costs of infection (Figure 1) were acting as strong constraints to selection, then the infection–condition relationship should be aligned in the same direction at the within‐ and among‐individual levels. In contrast, within‐individual constraints may not readily translate among individuals. For instance, animals in better condition or acquiring more food will be less limited by the amount of body resources than others (van Noordwijk & de Jong, 1986) and thus less subject to a resource allocation trade‐off between immunity and other functions (Seppälä, 2015). To account for this variation among hosts, the standard statistical approach of decomposing the phenotypic covariance between traits into within‐ and among‐individual components (Dingemanse & Dochtermann, 2013; Lynch & Walsh, 1998) has proven informative to detect a possible trade‐off (Careau & Wilson, 2017). Here, we apply such an approach to partition the condition–infection relationship (Figure 3). Within‐individual correlations (*r*_e_) based on repeated measurements are related to the relationship between changes in host response to infection and the change in condition. Hence the direction of *r*_e_ may support direct or indirect infection costs as previously described (Figure 3a,b). Among‐individual correlations (*r*_ind_) would represent the extent of both permanent environmental and genetic correlations between traits and may thus inform about potential constraints for selection. Hence, selection for host resistance should produce a positive *r*_ind_ (Figure 3c), whereas selection for host susceptibility should produce a negative *r*_ind_ (Figure 3d). Finally, a key interest of decomposing the condition–infection covariance is to identify scenarios where contrasting processes occur at the within‐ and among‐individual levels and may mask trade‐offs (Careau & Wilson, 2017). For instance, direct infection costs could predominate at the within‐individual level even in resistant animals (*r*_e_ < 0; Figure 3b), and mask among‐individual trade‐offs if for instance resistant animals tend to be in lower condition than others (*r*_ind_ > 0; Figure 3c).

To calculate the correlations among infection traits (FEC and ΔHE) and condition traits (BW, BFT, and MT) in each phase of infection, we fitted multivariate mixed‐effect models in ASReml 3.0. The previous univariate model [1] was thus extended to a multivariate model as follows:

(2) $$y_{\mathrm{kit}}=\beta_{0k}+\sum_{s=1}^{n_{k}}\beta_{\mathrm{ks}}x_{\text{ksit}}+{\mathrm{ind}}_{\mathrm{ki}}+e_{\mathrm{kit}},$$

where *y*_*kit*_ is the phenotypic observation of trait *k* for individual *i* at time *t* of infection and *β*_0_ and and *β*_*k*_ the *n*_*k*_ selected fixed effects associated with predictors of trait *k*. Random individual effects (ind_*ki*_) and random residual effects (*e*_*kit*_) were assumed to follow a multivariate normal distribution (MVN(0, Ω_ind_) and MVN(0, Ω_*e*_), respectively). The among‐individual (Ω_ind_) and within‐individual structures (Ω_*e*_) specified the variances of each trait *k* ($V_{{\mathrm{ind}}_{k}}$ and $V_{e_{k}}$) and all covariances for any pair of different traits *k* and *k'* (${\mathrm{COV}}_{{\mathrm{ind}}_{k,k\prime }}$ and ${\mathrm{COV}}_{e_{k,k\prime }}$) Specifically, considering the two infection traits and three condition traits, we modelled, for each infection stage, two 5 × 5 fully unstructured variance–covariance matrices:

$$\Omega_{\mathrm{ind}}=\left(\begin{array}{ccccc} V_{{\mathrm{ind}}_{\mathrm{FEC}}} & {\mathrm{COV}}_{{\mathrm{ind}}_{\mathrm{FEC},\mathrm{BW}}} & {\mathrm{COV}}_{{\mathrm{ind}}_{\mathrm{FEC},\mathrm{BW}}} & {\mathrm{COV}}_{{\mathrm{ind}}_{\mathrm{FEC},\mathrm{BFT}}} & {\mathrm{COV}}_{{\mathrm{ind}}_{\mathrm{FEC},\mathrm{MT}}}\\ {\mathrm{COV}}_{{\mathrm{ind}}_{\mathrm{FEC},∆\mathrm{HE}}} & V_{{\mathrm{ind}}_{∆\mathrm{HE}}} & {\mathrm{COV}}_{{\mathrm{ind}}_{∆\mathrm{HE},\mathrm{BW}}} & {\mathrm{COV}}_{{\mathrm{ind}}_{∆\mathrm{HE},\mathrm{BFT}}} & {\mathrm{COV}}_{{\mathrm{ind}}_{∆\mathrm{HE},\mathrm{MT}}}\\ {\mathrm{COV}}_{{\mathrm{ind}}_{\mathrm{FEC},\mathrm{BW}}} & {\mathrm{COV}}_{{\mathrm{ind}}_{∆\mathrm{HE},\mathrm{BW}}} & V_{{\mathrm{ind}}_{\mathrm{BW}}} & {\mathrm{COV}}_{{\mathrm{ind}}_{\mathrm{BW},\mathrm{BFT}}} & {\mathrm{COV}}_{{\mathrm{ind}}_{\mathrm{BW},\mathrm{MT}}}\\ {\mathrm{COV}}_{{\mathrm{ind}}_{\mathrm{FEC},\mathrm{BFT}}} & {\mathrm{COV}}_{{\mathrm{ind}}_{∆\mathrm{HE},\mathrm{BFT}}} & {\mathrm{COV}}_{{\mathrm{ind}}_{\mathrm{BW},\mathrm{BFT}}} & V_{{\mathrm{ind}}_{\mathrm{BFT}}} & {\mathrm{COV}}_{{\mathrm{ind}}_{\mathrm{BFT},\mathrm{MT}}}\\ {\mathrm{COV}}_{{\mathrm{ind}}_{\mathrm{FEC},\mathrm{MT}}} & {\mathrm{COV}}_{{\mathrm{ind}}_{∆\mathrm{HE},\mathrm{MT}}} & {\mathrm{COV}}_{{\mathrm{ind}}_{\mathrm{BW},\mathrm{MT}}} & {\mathrm{COV}}_{{\mathrm{ind}}_{\mathrm{BFT},\mathrm{MT}}} & V_{{\mathrm{ind}}_{\mathrm{MT}}} \end{array}\right)$$

and,

$$\Omega_{e}=\left(\begin{array}{ccccc} V_{e_{\mathrm{FEC}}} & {\mathrm{COV}}_{e_{\mathrm{FEC},\mathrm{BW}}} & {\mathrm{COV}}_{e_{\mathrm{FEC},\mathrm{BW}}} & {\mathrm{COV}}_{e_{\mathrm{FEC},\mathrm{BFT}}} & {\mathrm{COV}}_{e_{\mathrm{FEC},\mathrm{MT}}}\\ {\mathrm{COV}}_{e_{\mathrm{FEC},∆\mathrm{HE}}} & V_{e_{∆\mathrm{HE}}} & {\mathrm{COV}}_{e_{∆\mathrm{HE},\mathrm{BW}}} & {\mathrm{COV}}_{e_{∆\mathrm{HE},\mathrm{BFT}}} & {\mathrm{COV}}_{e_{∆\mathrm{HE},\mathrm{MT}}}\\ {\mathrm{COV}}_{e_{\mathrm{FEC},\mathrm{BW}}} & {\mathrm{COV}}_{e_{∆\mathrm{HE},\mathrm{BW}}} & V_{e_{\mathrm{BW}}} & {\mathrm{COV}}_{e_{\mathrm{BW},\mathrm{BFT}}} & {\mathrm{COV}}_{e_{\mathrm{BW},\mathrm{MT}}}\\ {\mathrm{COV}}_{e_{\mathrm{FEC},\mathrm{BFT}}} & {\mathrm{COV}}_{e_{∆\mathrm{HE},\mathrm{BFT}}} & {\mathrm{COV}}_{e_{\mathrm{BW},\mathrm{BFT}}} & V_{e_{\mathrm{BFT}}} & {\mathrm{COV}}_{e_{\mathrm{BFT},\mathrm{MT}}}\\ {\mathrm{COV}}_{e_{\mathrm{FEC},\mathrm{MT}}} & {\mathrm{COV}}_{e_{∆\mathrm{HE},\mathrm{MT}}} & {\mathrm{COV}}_{e_{\mathrm{BW},\mathrm{MT}}} & {\mathrm{COV}}_{e_{\mathrm{BFT},\mathrm{MT}}} & V_{e_{\mathrm{MT}}} \end{array}\right)$$

Moreover, only during PP1 and PP2, there were enough data to fit line‐specific variance–covariances structures (i.e. $\Omega_{{\mathrm{ind}}_{R}}$, $\Omega_{{\mathrm{ind}}_{S}}$, $\Omega_{e_{R}}$, $\Omega_{e_{S}}$), as proposed in Figure 3.

The correlations between each pair of traits *k* and *k*' among‐ ($r_{{\mathrm{ind}}_{k,k^{\prime }}}$) and within‐individual ($r_{e_{k,k^{\prime }}}$) were calculated from the estimates variances covariances:

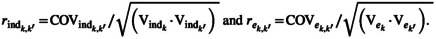




We tested the significance of a particular correlation *r*_*k*,*k'*_ using likelihood ratio test (likelihood comparison of the full model estimating all covariance terms to the model where *r*_*k*,*k'*_ is constrained to zero). During PP1 and PP2, differences between line‐specific correlations were also tested ($r_{{\mathrm{ind}}_{k,k\prime R}}$ vs. $r_{{\mathrm{ind}}_{k,k\prime S}}$ and $r_{e_{k,k\prime R}}$ vs. $r_{e_{k,k\prime S}}$). This was done by comparing the likelihood of the model fitting two line‐specific correlations with the likelihood of the model including a same correlation for both lines.

## 3 RESULTS

### 3.1 Infection traits of resistant and susceptible lines across successive infections

#### 3.1.1 Lamb stage

Infection traits during the lamb phase were directly related to selection responses. All 91 growing female sheep of 4–5 months of age excreted parasite eggs after their first exposure to *H. contortus* (single‐dose of 3500 L3 per lamb). The following infection was based on higher dose (10,000 L3 per lamb) but resulted in lower levels of egg excretion: most lambs from the R line even had no excretion at all (32 out of 51) but also some from the S line (7 out of 40). However, the difference in FEC (back‐transformation of log[FEC + 1]) became clearer in this second infection (R [mean ± *SE*]: 8 ± 1.55 eggs per gram; S: 123 ± 1.6 eggs per gram). Despite the large number of null values of FEC, diagnostic plots indicated that model fitting was satisfactory (Figure S1). High correlations between phenotypic measures and individual EBVs for parasite resistance (i.e. the average EBV from the two parents) also showed that genetic variation in this trait was strongly expressed after the first exposure to the parasite (Figure S2). The divergence between R and S lambs was also clear from the differential loss in blood haematocrit from day 21 onwards (Table S1). At the end of the infection (day 35), ΔHE was three times larger in the S line (ΔHE_S_ = + 3.28 ± 0.53%) compared to the R line (ΔHE_R_ = + 1.04 ± 0.47%).

#### 3.1.2 Adult reproductive stages

**FIGURE 4:**
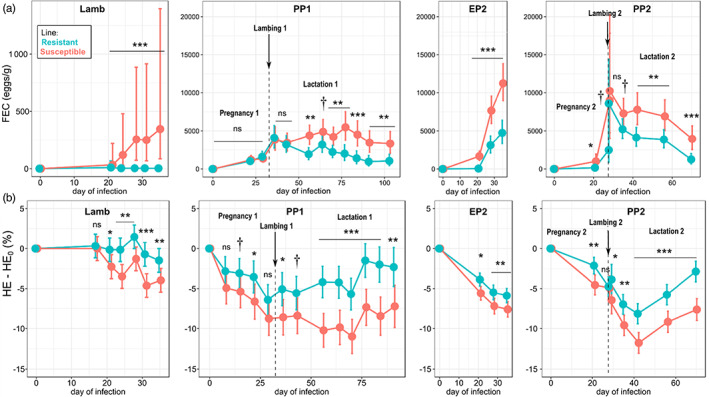
Fecal egg count (FEC; upper panels) and the change in blood haematocrit (HE) compared to the individual initial level (HE_0_) (bottom panels), in response to successive infections in female sheep divergently selected on resistance to *Haemonchus contortus*. Circles are adjusted means with their error bars representing 95% confidence interval. See details about stages and infections in Figure 2. Asterisks indicate statistical differences between lines (†: *p* < 0.1; *: *p* < 0.05; **: *p* < 0.01; ***: *p* < 0.001). Note the different y‐axis scale for FEC during the lamb phase compared to the three adult reproductive phases to enhance visibility

Across reproductive stages, the difference in line responses to the three phases of infection supported a genetically based divergence in parasite resistance (Tables S2–S4). A line effect on FEC (transformed) was detected during lactation of PP1 (likelihood ratio test of the model including ‘line’ (without interactions) vs. the model excluding ‘line’: χ_(1)_ = 8.86, *p* = 0.003), EP2 (χ_(1)_ = 35.9, *p* < 0.001), and PP2 (gestation: χ_(1)_ = 11.0, *p* < 0.001; lactation::χ_(1)_ = 13.2, *p* < 0.001). However this effect was largely attenuated shortly before lambing and for about 1 month in lactation (as observed from the model including the interaction between line and day of infection; Figure 4). Interestingly, this reduction in the FEC difference between lines followed a similar pattern during PP1 and PP2, although those two phases of infection had a different mode. In the S line, FEC continued to increase about 7 weeks after lambing during PP1 (as trickle infection still occurred 3 weeks after lambing and was bringing novel cohorts of worms), whereas it declined after lambing during the single‐dose infection of PP2 (reflecting the decline in the single cohort of worms whose mean life expectancy is about 50 days). In contrast, the R line always reached maximum FEC around lambing and decreased afterwards. Consistently with those changes in FEC, the R line started to recover blood haematocrit sooner than the S line (Figure 4).

### 3.2 Condition traits of resistant and susceptible lines across successive infections

#### 3.2.1 Growing lambs

**FIGURE 5:**
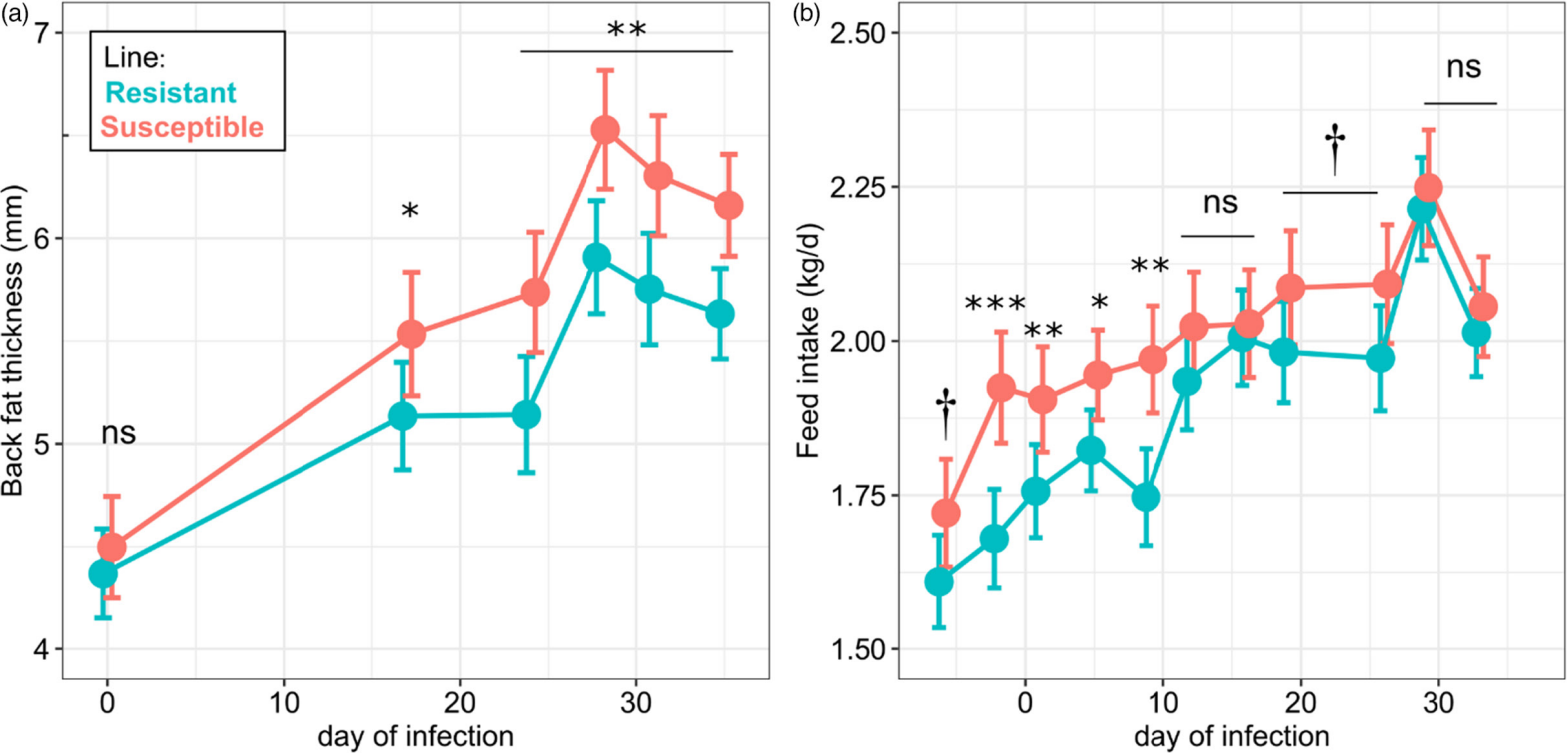
Backfat thickness (a) and voluntary feed intake (b) during parasitic infection of growing female lambs from lines divergently selected on resistance to *Haemonchus contortus*. Circles are adjusted means with their error bars representing 95% confidence interval. Asterisks indicate statistical differences between lines (†: *p* < 0.1; *: *p* < 0.05; **: *p* < 0.01; ***: *p* < 0.001)

Despite the infection, lambs of both lines grew rapidly (about 300 g/day during the 5 weeks of the second infection) as concentrate feed was provided *ad libitum*. The low parasitic load in the R line did not translate into greater gain in body weight compared to the S line. Conversely, R lambs ate less then S lambs (*β*_R–S_ = −102 g/day, *t*_86_ = −2.4, *p* = 0.017) and deposited less fat on their back (Table S1). Most of these differences seemed to be generated during the first 2 weeks of infection, which coincides with the prepatent period of *H. contortus* (Figure 5). However, these differences in feed intake between lines do not necessarily indicate different responses to infection (e.g. possibly a more pronounced parasite‐induced anorexia in the R line) as the S line also had a larger feed intake few days prior to infection (Figure 5b).

#### 3.2.2 Reproductive ewes

Later on, R and S ewes infected during reproduction no longer exhibited a difference in backfat thickness (Figure S3). Their body weight did not differ throughout pregnancy and lactation (Table S2–S4). Only muscle thickness tended to be lower in the S line during lactation 1 (*β*_R–S_ = 0.79 mm, *t*_47_ = 1.88, *p* = 0.066) and 2 (*β*_R–S_ = 0.929 mm, *t*_46_ = 1.917, *p* = 0.06). This, together with the larger ΔHE in the S line, tend to indicate infection costs involving protein metabolism during reproduction.

Susceptibility to parasite also affected milk composition (Table S5). In milk sampled 1 month after lambing, protein content was lower in the S line during PP1 (*β*_R–S_ = 2.9 g/L, *t*_46_ = 2.1, *p* = 0.042) and during PP2 (*β*_R–S_ = 3.4 g/L, *t*_46_ = 2.4, *p* = 0.021). Fat content was also affected but only during PP2 (*β*_R–S_ = 11.3 g/L, *t*_46_ = 2.4, *p* = 0.024). However, those differences in milk quality did not translate into different lamb growth rates (Figure S4.1). During PP2, twins born from the S ewes were even growing faster than those from the R ewes but also weighed less at birth (Table S6; Figure S4.2).

### 3.3 Effect of dietary protein restriction on infection traits and condition traits during the periparturient period

During PP1, protein diet restriction had no effect on FEC (Table S2). In contrast, it exacerbated the loss in haematocrit, especially in the S line (differential in ΔHE between low‐protein and high‐protein during pregnancy = 11.27, *t*_47_ = 5.76, *p* < 0.001). Under the low‐protein diet, ΔHE was almost twice as large in the S line as in the R line (R: 6.76 ± 1.26% vs. S: 12.1 ± 1.42%). In contrast, lines had similar ΔHE when they consumed the high‐protein diet (R: 1.16 ± 1.26% vs. S: 0.79 ± 1.36%). This interaction between line and diet was maintained during lactation of PP1 (Table S2). During PP2, the same high‐protein diet provided to all ewes did not prevent a larger ΔHE in S ewes compared with R ones (Figure 2, Table S4), suggesting that the high‐protein diet was apparently not compensating for the genetic divergence in parasite resistance between lines. However, in contrast to PP1 in which ewes were trickle‐infected with 1000 *H. contortu*s L3 weekly, PP2 infection was based on a heavy initial parasite load as a result of a single bolus infection of 10,000 *H. contortus* L3, which may have led to some saturation of the host immune system.

Protein restriction had clear negative effects on condition traits, regardless of the lines (Figure S3). Lactating ewes fed with the low‐protein diet were 2.87 ± 1.43 kg lighter than those fed with the high‐protein diet (Table S2, Figure S1). Backfat and muscle thickness were also reduced during pregnancy of PP1 (BFT: 4.71 ± 0.15 mm for high‐protein vs. 4.33 ± 0.16 mm for low‐protein; MT: 22.5 ± 0.26 mm for high‐protein vs. 21.4 ± 0.26 mm for low‐protein), but no longer after lambing. At birth, lambs born from protein‐restricted ewes were about 10% lighter than those born from unrestricted ewes (Table S5). During lactation, protein‐restricted ewes had lower milk fat content (*β*_HProt–LProt_ = 17.1 g/L, *t*_46_ = 3.89, *p* < 0.001); however, only lambs from the S ewes were growing slower (Table S6; Figure S4.1). Overall, it seemed thus that the protein content of the diet was only limiting for the expression of certain benefits of parasite resistance such as ewe haematocrit and growth of their lambs.

### 3.4 The condition–infection relationship

**FIGURE 6:**
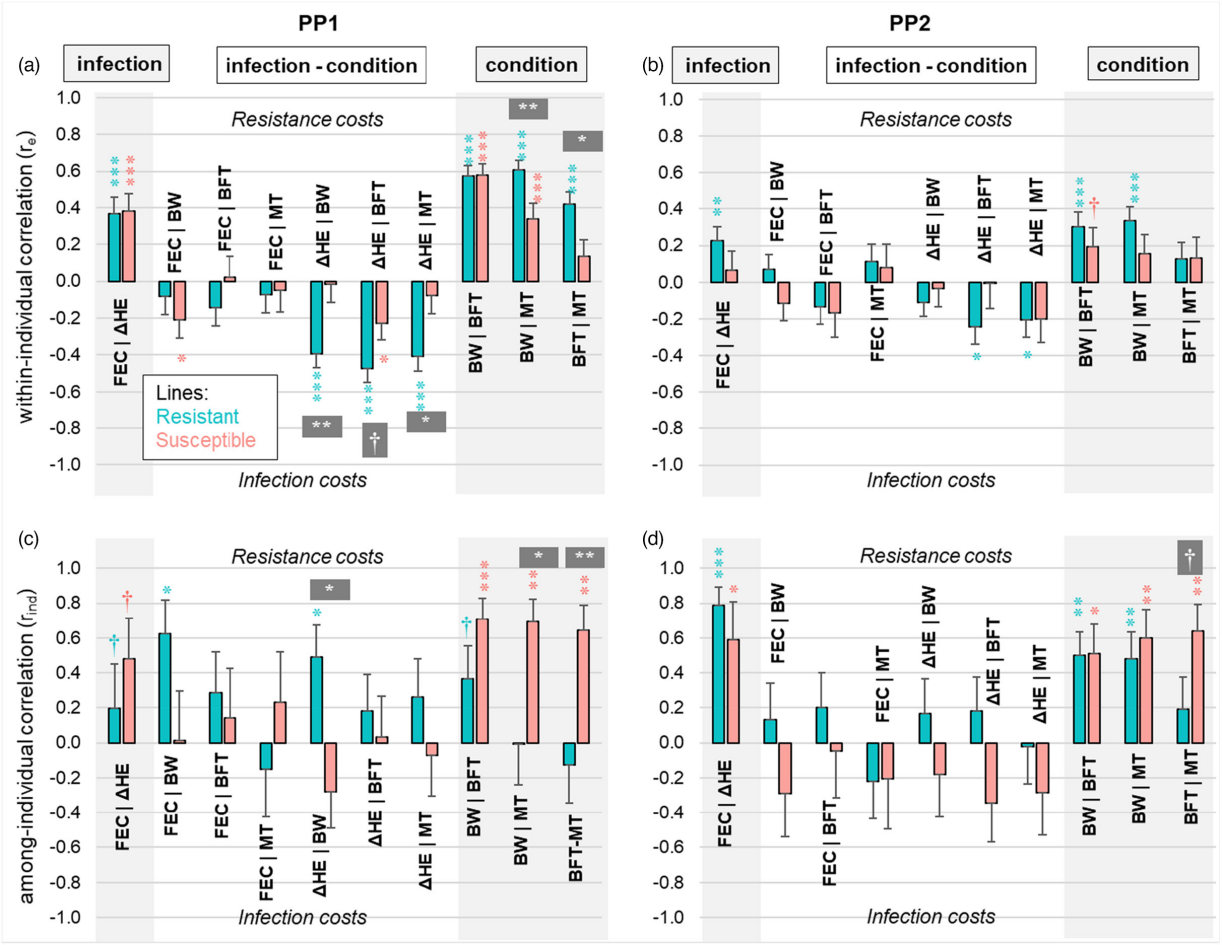
Correlations among infection traits and condition traits in females from sheep lines divergently selected for resistance to *Haemonchus contortus* during peripartum infections at 1year of age (PP1) and at 2 years of age (PP2). Correlations are decomposed into within‐individual (a and b) and among‐individual (c and d), as proposed in Figure 3. Hence positive correlations indicate that resistance costs on condition are higher than direct infection costs, while the opposite corresponds to negative correlations. Error bars represent correlation standard errors and the level of statistical significance (null hypothesis is zero correlation). Statistical significance of the difference between line‐specific correlations is represented in grey boxes. BFT, back fat thickness; BW, body weight; FEC, fecal egg count; ΔHE, loss in blood haematocrit; MT, muscle thickness

Multivariate models applied to PP1 and to PP2 (pregnancy and lactation) allowed to disentangle the condition–infection relationship in each line (Figure 6; Tables S9 and S11), as proposed (Figure 3). This covariance decomposition revealed three main outcomes: first, at the within‐individual level (Figure 6a,b), consistent positive r_e_s were observed between the two infection traits (FEC and ΔHE) and among the three condition traits (BW, BFT, and MT), whereas most r_e_s between these two kinds of traits (i.e. condition–infection *r*_*e*_s) were null or negative, in accordance with direct infection costs (Figure 3b). Those r_e_s were generally stronger for the R line than for the S line, particularly *r*_*e*_ between ΔHE and condition traits for which significant lines differences were detected. Second, at the among‐individual level (Figure 6c,d), the strong positive *r*_ind_s that were expected within each type of trait (i.e. infection *r*_ind_s and condition *r*_ind_s) were only observed for the S line, whereas some *r*_ind_s, notably among condition traits during PP1, did not meet this expectation in the R line. In other words, we found an indication that the among‐individual covariation in BW and composition (BFT, MT) is sensitive to host resistance during peripartum infection. Third, in the R line, we further found that most condition‐infection *r*_ind_s were null or positive (even though the only significant *r*_ind_ was between infection traits and BW during PP1 [Figure 6C]), which provided support for resistance costs (Figure 3C). In contrast, no clear trend was observed for the S line, although the whole negative (yet non‐significant) values of *r*_ind_ during PP2 may point to predominant infection costs in this line. Exact values for estimated correlations are shown in Tables S9–S12. Finally, as the set of selected fixed effects were sometimes different between traits (Table 2), we checked to what extent this affected the estimates of *r*_*e*_ and *r*_ind_. We re‐estimated these correlations using the same fixed structure for all traits and found that their values remained largely unchanged compared with those obtained using the selected fixed effects for each trait (Table S12).

## 4 DISCUSSION

In this study, we found that divergent selection based on responses to artificial infection with *H. contortus* in young sheep was also effective in adult females, except around parturition, where our observations indicated a classic periparturient relaxation of immunity (PPRI), both in the resistant and in the susceptible line. Although we found some support for a cost of parasite resistance on condition during growth, dietary responses in condition during peripartum infection seemed phenotypically independent of ewes' genetic background. At first glance, our results were thus at odds with a role of body resource allocation during the PPRI but consistent with a resource‐independent constraint. However, decomposing the condition–infection relationship per line revealed that selection for parasite resistance actually incurred a cost on body weight among ewes around their first lambing. Thus, both a physiological constraint related to PPRI and a trade‐off between host resistance and condition may be involved during reproduction in young female sheep. In light of their relatively small effect, it seems nevertheless unlikely that those limits are strongly opposed to directional selection for host resistance in nutrient‐rich environments.

The female reproductive constraint of the PPRI is a priori deeply rooted in mammalian evolution as this is a common feature of several species (Houdijk et al., 2001). Sheep exposed to gastrointestinal nematodes provide a relevant model system for such constraint as the PPRI is of real consequence for animal performance and for the efficiency of livestock production (Beasley et al., 2010; Hayward et al., 2019; O'Sullivan & Donald, 1973). Our results further indicate that the PPRI is relatively insensitive to selection for resistance (or susceptibility) to one of the most common and pathogenic parasites in sheep. In a similar divergent selection experiment for lamb resistance against artificial infection with *H. contortus* in the Merino breed (Woolaston et al., 1990), consistent results were obtained in ewes exposed to natural multi‐species gastrointestinal infection (Woolaston, 1992). As in our study, the periparturient rise in FEC was still observed in the R line, although its duration and magnitude were attenuated compared to the S line (Kahn et al., 2003). In Romney sheep lines divergently selected on resistance to natural nematode infections (Morris et al., 1997), the FEC divergence selected in lambs was maintained at about 70% in lactating ewes 1–2 months after lambing (Morris et al., 1998), which is consistent with the post‐lambing delay to recover genetically based resistance in our study. Evidence from those different selection experiments and others (Morris et al., 2005 in Perrendale sheep) then suggests that the persistence of the PPRI against selection for resistance to parasites unlikely reflects an insufficient selection response. In our case, study animals were only from the second generation of divergent selection for parasite resistance, but the genetic divergence between lines was large due to the high selection intensity and the relatively high FEC heritability (c.a. 0.3–0.55) obtained under controlled host environment and conditions of infection (Sallé et al., 2021). We performed a supplementary analysis of the correlations between stage‐specific responses to infection, which further indicated that FEC was highly repeatable across stage‐specific infections (correlation between stages >0.5 in general; Appendix S2 and Table S7). Our results were thus globally consistent with the high genetic correlations observed between FEC measures at different ages in other sheep populations (Bishop & Stear, 2001; Brown & Fogarty, 2017; Goldberg et al., 2012; Notter et al., 2018; Pollott et al., 2004). Overall, there is little chance that periparturient limits to selection for parasite resistance stem from a lack of genetic variation at this stage (Bishop & Stear, 2001; Goldberg et al., 2012).

While the nutritional basis of the PPRI is largely acknowledged (Coop & Kyriazakis, 1999), our results of protein restriction did not support the hypothesis that the immunosuppressive effects of reproduction essentially depend on nutrient availability (i.e. are non‐obligatory), as considered in the ‘facultative regulation hypothesis’ (French et al., 2007; Rauw, 2012). In domestic sheep, a large body of evidence exists that protein supplementation reduces the magnitude of the PPRI (Beasley et al., 2012; Coop & Kyriazakis, 2001; Donaldson et al., 1998; Houdijk et al., 2001; Kahn et al., 2003). This is in line with the view that the PPRI results from scarce protein allocation to gestation or lactation, at the expense of parasite‐specific components of maternal immunity (Coop & Kyriazakis, 1999). However, the benefits of protein nutrition in terms of PPRI attenuation appeared relatively small in genetically resistant ewes compared with more susceptible ewes (Kahn et al., 2003; Kidane et al., 2010). Accordingly, we observed larger benefits of protein supplementation in terms of ΔHE in the S line than in the R line during PP1. In contrast, the lack of dietary effect on FEC even in the S line may seem surprising. Yet, the larger benefits of protein supplementation on PPRI are usually expected in grazing conditions of low‐quality pasture and when ewes are in poor condition (Macarthur et al., 2013; Valderrábano & Uriarte, 2003), whereas animals of our experiments were exclusively indoor and in good condition prior to the different infections.

Similar to other studies in mammals, our results point to a regulation of immunity during the PPRI that is independent of nutrient availability in the environment (Speakman, 2008 (small mammals); Albery et al., 2020 (red deer in the wild); Trillmich et al., 2020 (Guinea pig in the laboratory)). However, the exact nature of this regulation remains unclear as none of the different mechanisms that could explain immunosuppression during gestation and/or lactation has gained acceptance so far (Barger, 1993; Beasley et al., 2012; Jeffcoate et al., 1992). Here, the failure to detect a significant difference in FEC between lines around lambing suggests that PPRI imposes an overriding constraint on the expression of genetic resistance to parasites. To go further into the proximate mechanisms underlying the breakdown of immunity in female mammals, our sheep lines then provide a valuable system model. Yet, an important prerequisite will be to clarify the immunogenetic basis of the selected resistance to *H. contortus*. A previous study of our host–pathogen system but with non‐selected animals has clearly supported a Th2‐oriented immune response to *H. contortus* (Lacroux et al., 2006), as seen for other gastro‐intestinal nematode infection (Allen & Maizels, 2011). However, an accurate immune marker of the selected resistance against *H. contortus* may be particularly challenging to identify considering that the timing of immune response, rather than its magnitude, can be the most determinant aspect (Hamie et al., 2019). Moreover, no major effect QTL has been identified so far in our lines (Sallé et al., 2012, 2021). Despite the seemingly high degree of redundancy in immunological responses to *H. contortus*, selection for parasite resistance has apparently not promoted a mechanism circumventing PPRI.

Under abundant and nutrient‐rich environments, an obligate regulation of immunity may be consistent with predictable changes in host resource allocation (French et al., 2007; Trillmich et al., 2020), in accordance with the concept of genetically driven changes in nutrient partitioning established in reproducing females of livestock species (Bauman & Currie, 1980; Chilliard, 1992; Friggens et al., 2004). In other words, females are thought to anticipate the high nutrient demand for reproductive expenditure during the periparturient period by systemically suppressing their immune response. This hypothesis, however, critically depends upon the magnitude of a nutritional cost of resistance to *H. contortus*. As other studies (Greer, 2008; Liu, Smith, Briegel, et al., 2005; Liu, Smith, Karlsson, et al., 2005, as well preliminary observations in the R line (Sallé et al., 2021)), our data support a cost of acquiring resistance during lamb growth (here expressed as a reduction in back fat deposition). As the reduced rate of back fat deposition was ambiguously associated with a reduction in feed intake, we could not, however, infer if it was reflecting a direct infection cost (e.g. a decrease in nutritional supply due to parasite‐induced anorexia) or an indirect cost (e.g. a mobilisation of specific nutrients to fulfil the induced requirements of the immune response (Kyriazakis et al., 1998)). In addition, during the periparturient period, female feed intake is often depressed, especially in sheep, which may further promote a competitive use of body resources among functions. In females from the R line, we detected costs on body weight during the first periparturient period, while growth was still ongoing and reproductive effort was high, whereas no cost was detected during the second periparturient period when ewes were fully grown (even though the infectious challenge was stronger). Early costs of host resistance on body weight together with the breakdown of among‐individual correlations among condition traits during first reproduction suggest that despite the PPRI, selection for host resistance favours the reallocation of body resources from growth to immunity and thus alters the priorities of nutrient allocation (Coop & Kyriazakis, 1999).

Contrary to our expectation, we found that within‐individual correlations describing the condition‐infection relationship during the periparturient period were supporting direct infection costs in both lines, which were in some cases stronger for the R line than for the S line. Moreover, condition traits of the S line were mostly identical to those of the R line during infections, even when a large difference in parasite burden was observed. This seems to concur with other results from divergent selection on parasite resistance in sheep showing that selection for susceptibility may favour compensatory mechanisms that involve a lesser degree of anorexia, an improved feed conversion efficiency, or an increased protein synthesis (Doyle et al., 2011; Liu, Smith, Briegel, et al., 2005). As mechanisms involved in host resistance can trade‐off against those involved in host tolerance (Vale et al., 2016), further research in our lines should look at the correlated responses in host tolerance. Although our framework (Figure 3) relying on a multivariate random intercept model had merit to unmask resistance costs among interrelated condition traits, studying the potential trade‐off between resistance and tolerance would require more sophisticated approaches. For instance, individual variation in tolerance could be considered by modelling the within‐individual change in condition according to infection severity using random regression (Hayward et al., 2014b). Moreover, within‐host infection dynamics certainly exhibit a high degree of non‐linearity and asynchrony (Lough et al., 2015), so positive and negative condition‐infection relationships probably occur over the course of infection. Such complex dynamics could be accounted, for instance, through the analysis of two‐dimensional trajectories of individuals' condition‐infection dynamics (Doeschl‐Wilson et al., 2012; Lough et al., 2015).

This experimental study provides rare evidence for costs of host resistance on condition under an obligate constraint of female reproduction. It offers insights into one of the numerous potential mechanisms through which the condition‐infection relationship can affect population responses to selection for resistance. Despite attenuated selection responses during the periparturient period, we have shown subtle costs of genetic resistance to parasites on host condition in primiparous females. Our results seem overall consistent with the view that livestock resistance to gastro‐intestinal parasitism entails a relatively short‐term diversion of nutrients from biosynthesis to immunological processes in order to provide a long‐term advantage (Greer, 2008). The small costs on condition and the transient reproductive constraint on host resistance that we assessed are unlikely to represent strong limits to selection in relatively controlled and nutrient‐rich environments. However, effective parasite control strategies on‐farm would probably require management practices that compensate for the temporal inefficacy of genetic resistance during the periparturient period. Moreover, estimation of the genetic correlations between condition and infection traits at first mating will ascertain the genetic relationships between those two kinds of traits that we found positively linked. This would require large‐scale quantitative genetic studies that account for direct and indirect infection costs at the within‐ and among‐individual levels – which might be extremely challenging, yet critical given the large variation in genetic correlation observed among studies (Greer, 2008; Mucha et al., 2022). From a theoretical perspective, considering the costs of genetic host resistance under the constraints of female reproduction strengthens the mechanistic basis of the classic evolutionary theory of parasite mediated‐selection (Sheldon & Verhulst, 1996). So far, the theory has emphasized the incurrence of direct condition costs of infection following the immunosuppressive effects of reproduction, rather than the indirect infection costs associated with host resistance (Leivesley et al., 2019). Although some evidence exists for a genetic antagonism between host resistance and reproductive effort both in domestic populations (Bishop & Stear, 2001; Notter et al., 2018) and in the wild (Hayward et al., 2014a), the mediation of such trade‐off through condition costs is yet to be explored.

## CONFLICT OF INTEREST

The authors declare no conflicts of interest.

## Supporting information


Figure S1

Figure S2

Figure S3

Figure S4

Table S1–S12

Appendix S1

Appendix S2

## Data Availability

Data for this study are available at: https://figshare.com/s/5368d802985f84ef576a.
