# Supplementary material for: The cost of host genetic resistance on body condition: Evidence from divergently selected sheep
Source: Evol Appl. 2022 Jul 12;15(9):1374–89. doi: 10.1111/eva.13442 (PMC9488686; doi:10.1111/eva.13442)
Supplement: Supplementary file 1 — Figure S1 [file EVA-15-1374-s006.docx]

**Figures S1. Diagnostic plots of linear mixed model of transformed measures of fecal egg count during the different infections**


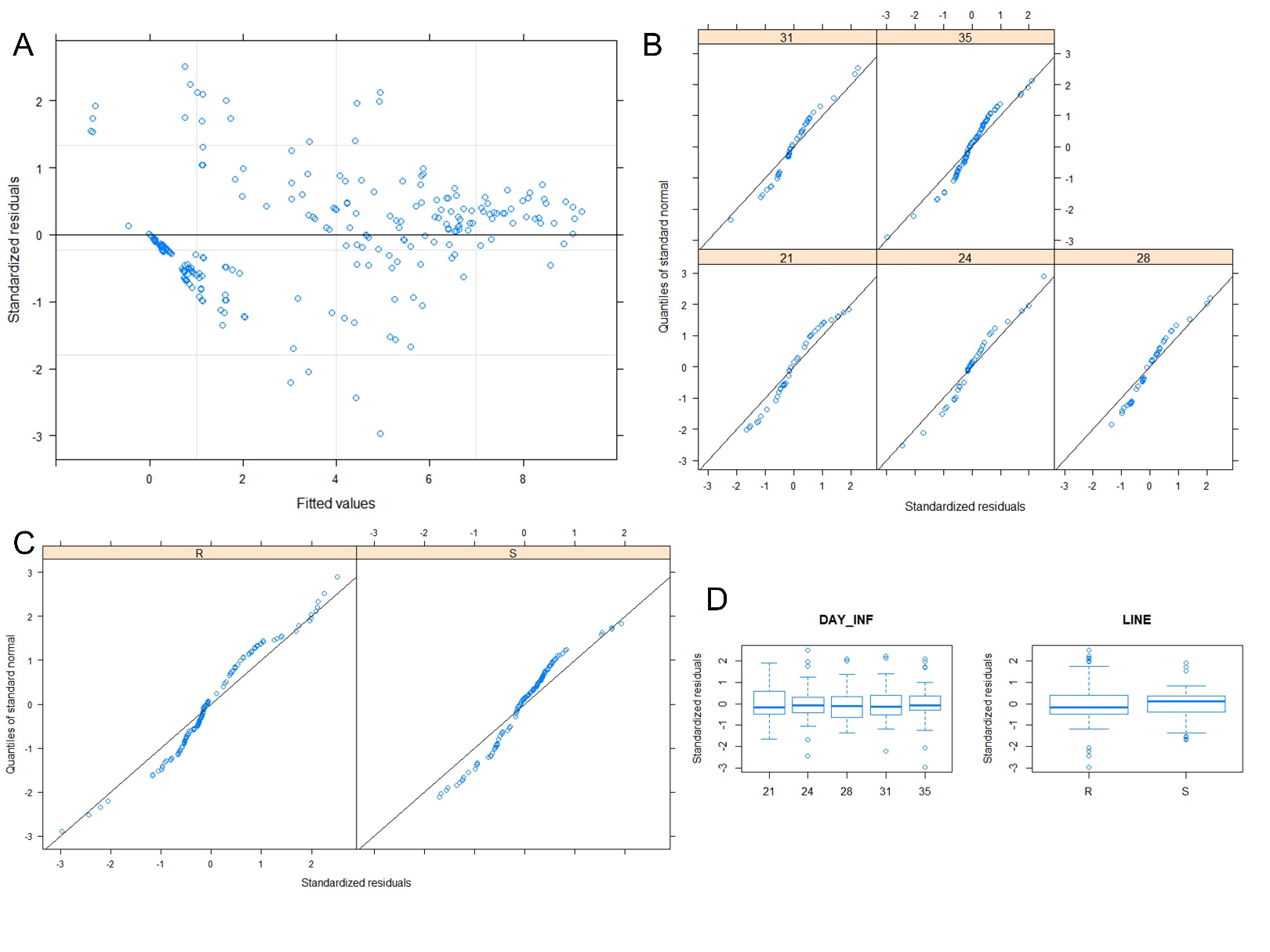


**Figure S1.1: Diagnostic plots of the linear mixed model of log(FEC + 1) during infection in the ‘Lamb’ phase** (detailed in Table S1): (A) standardized residuals vs. fitted values; (B) normal Q-Q plot according to the day of infection; (C) normal Q-Q plot according to the line; (D) distribution of standardized residuals according to the day of infection (variance heterogeneity accounted for in the model, Table S1) and to the line.


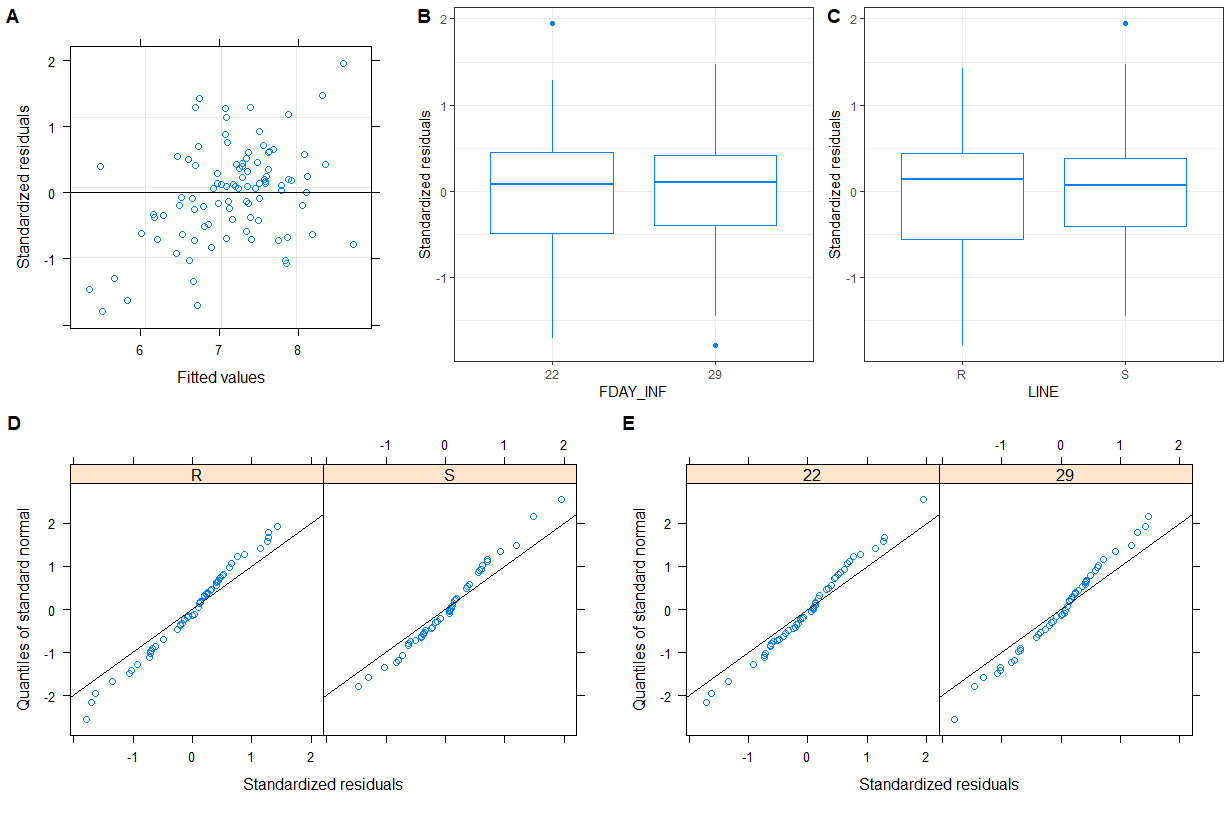


**Figure S1.2: Diagnostic plots of the linear mixed model of log(FEC + 1) during infection in pregnancy during the ‘PP1’ phase** (detailed in Table S2): (A) standardized residuals vs. fitted values; (B) distribution of standardized residuals according to the day of infection, (C) or to the line; (D) normal Q-Q plot according to the line; (E) normal Q-Q plot according to the day of infection.


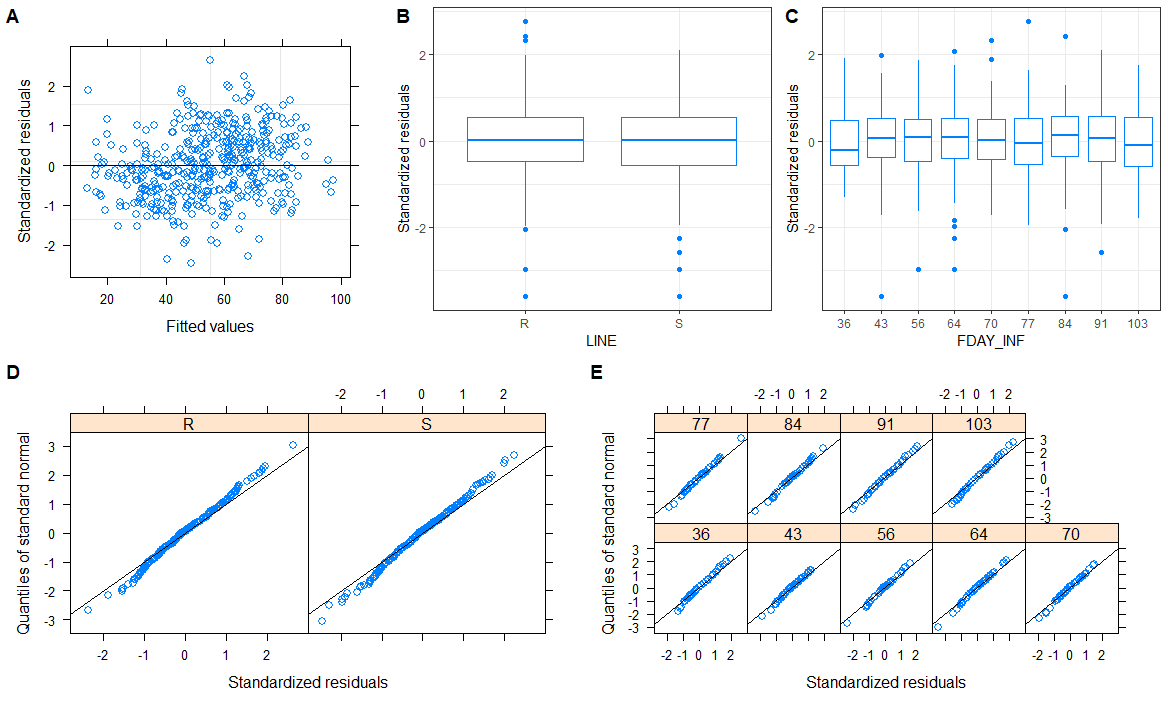


**Figure S1.3**: **Diagnostic plots of the linear mixed model of FEC^0.5^ during infection in lactation during the ‘PP1’ phase** (detailed in Table S2): (A) standardized residuals vs. fitted values; distribution of standardized residuals: (B) according to the day of infection (variance heterogeneity accounted for in the model, Table S2) or (C) according to the line; (D) normal Q-Q plot according to the line; (E) normal Q-Q plot according to the day of infection.


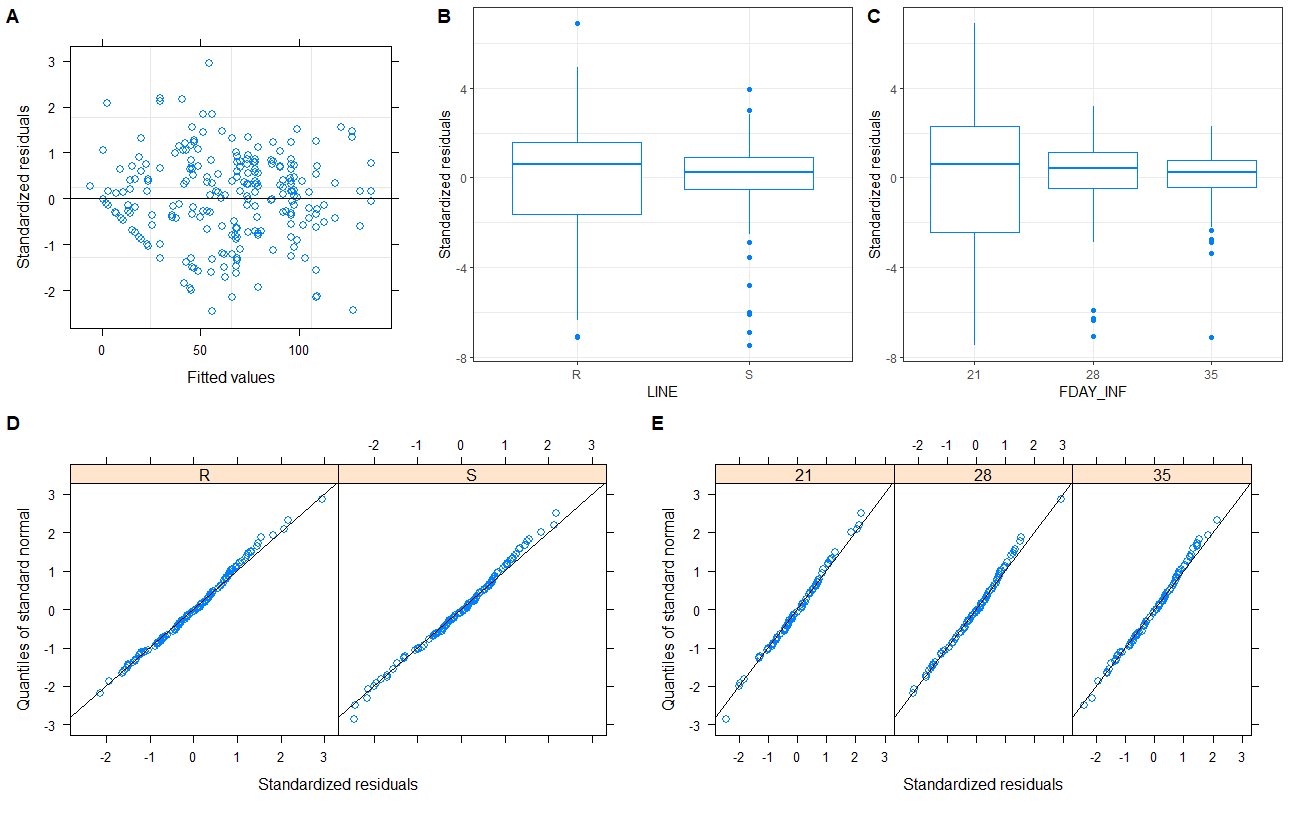


**Figure S1.3: Diagnostic plots of the linear mixed model of FEC^0.5^ during infection in the ‘EP2’ phase** (detailed in Table S3): (A) standardized residuals vs. fitted values; distribution of standardized residuals: (B) according to the day of infection (variance heterogeneity accounted for in the model, Table S3), (C) according to the line; (D) normal Q-Q plot according to the line; (E) normal Q-Q plot according to the day of infection.


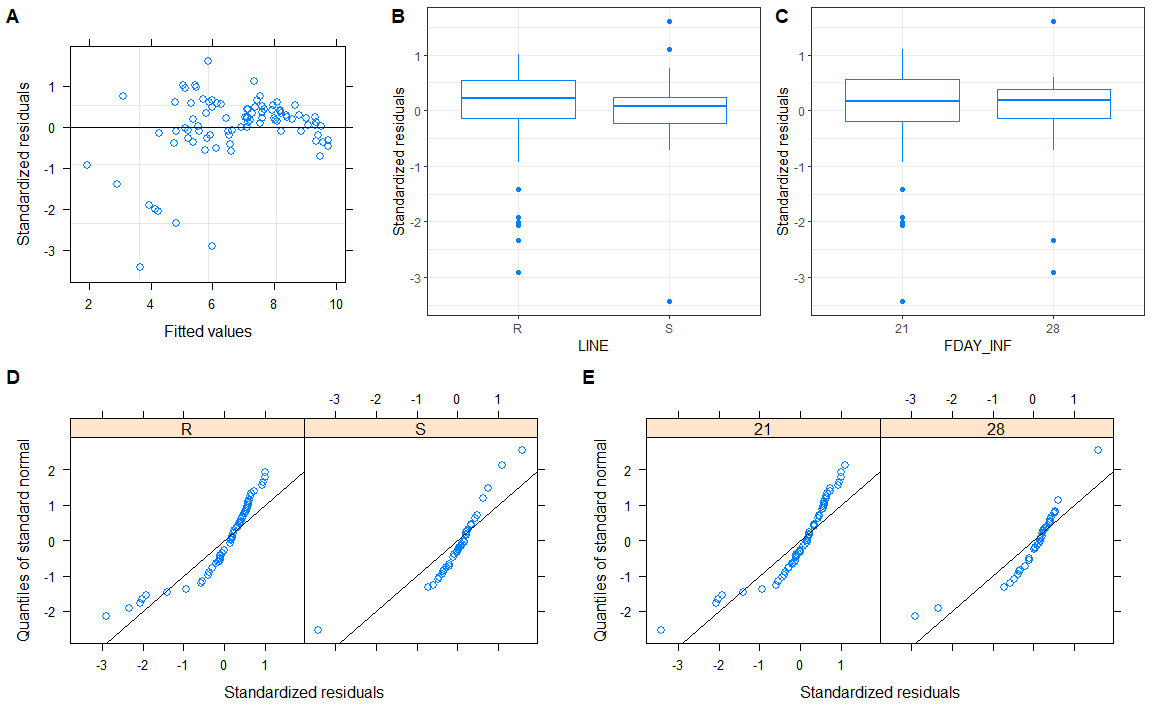


**Figure S1.4: Diagnostic plots of the linear mixed model of log(FEC + 1) during infection in pregnancy during the ‘PP2’ phase** (detailed in Table S4): (A) standardized residuals vs. fitted values; distribution of standardized residuals: (B) according to the day of infection, (C) according to the line (variance heterogeneity accounted for in the model, Table S4); (D) normal Q-Q plot according to the line; (E) normal Q-Q plot according to the day of infection.


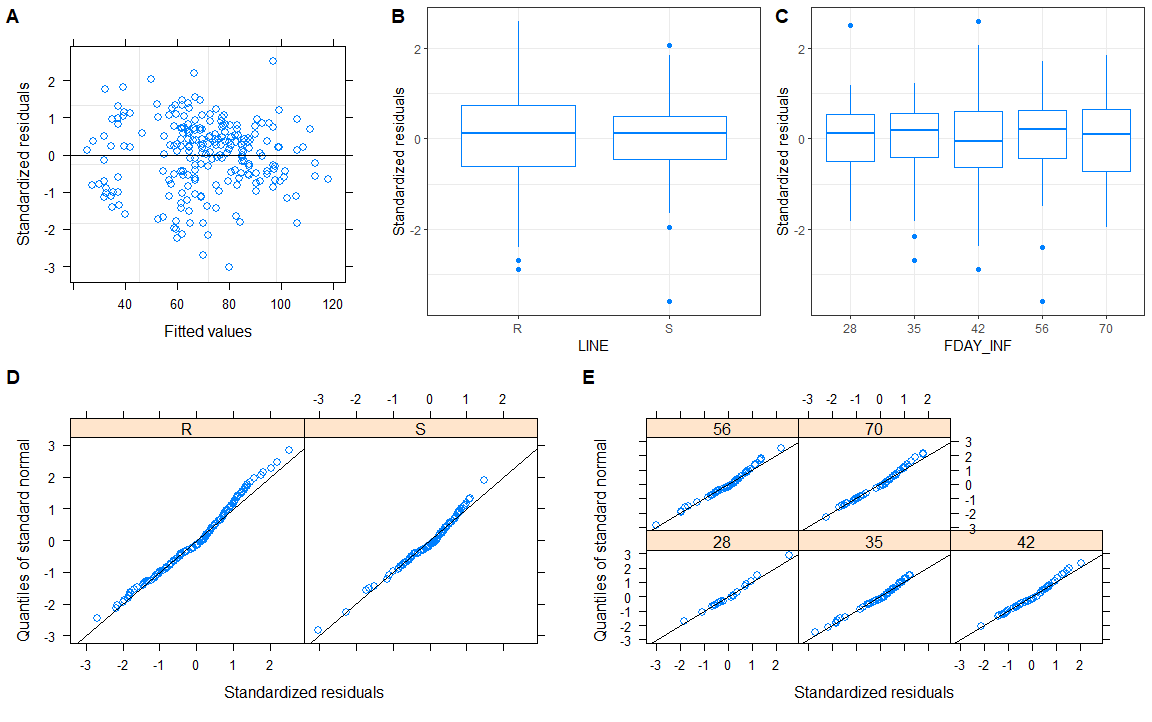


**Figure S1.5: Diagnostic plots of the linear mixed model of FEC^0.5^ during infection in lactation the ‘PP2’ phase** (detailed in Table S4): (A) standardized residuals vs. fitted values; distribution of standardized residuals: (B) according to the day of infection (variance heterogeneity accounted for in the model, Table S3), (C) according to the line; (D) normal Q-Q plot according to the line; (E) normal Q-Q plot according to the day of infection.
