# Supplementary material for: The cost of host genetic resistance on body condition: Evidence from divergently selected sheep
Source: Evol Appl. 2022 Jul 12;15(9):1374–89. doi: 10.1111/eva.13442 (PMC9488686; doi:10.1111/eva.13442)
Supplement: Supplementary file 2 — Figure S2 [file EVA-15-1374-s005.docx]

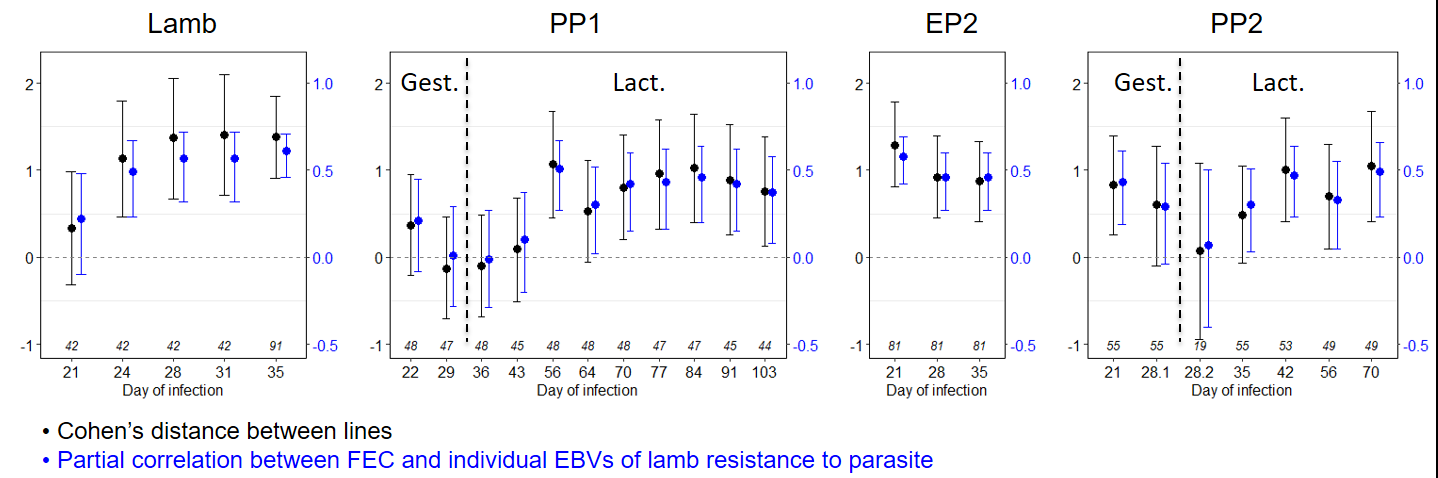


**Figure S2**: Fecal egg count (FEC) Cohen’s distance between lines of female sheep divergently selected on resistance to *H. Contortus*, and partial correlation between FEC and estimated breeding values (EBV) of lamb resistance to parasites. Error bars representing 95% confidence interval. See details about stages and infections in Figure 2.
