# Supplementary material for: The cost of host genetic resistance on body condition: Evidence from divergently selected sheep
Source: Evol Appl. 2022 Jul 12;15(9):1374–89. doi: 10.1111/eva.13442 (PMC9488686; doi:10.1111/eva.13442)
Supplement: Supplementary file 3 — Figure S3 [file EVA-15-1374-s003.docx]

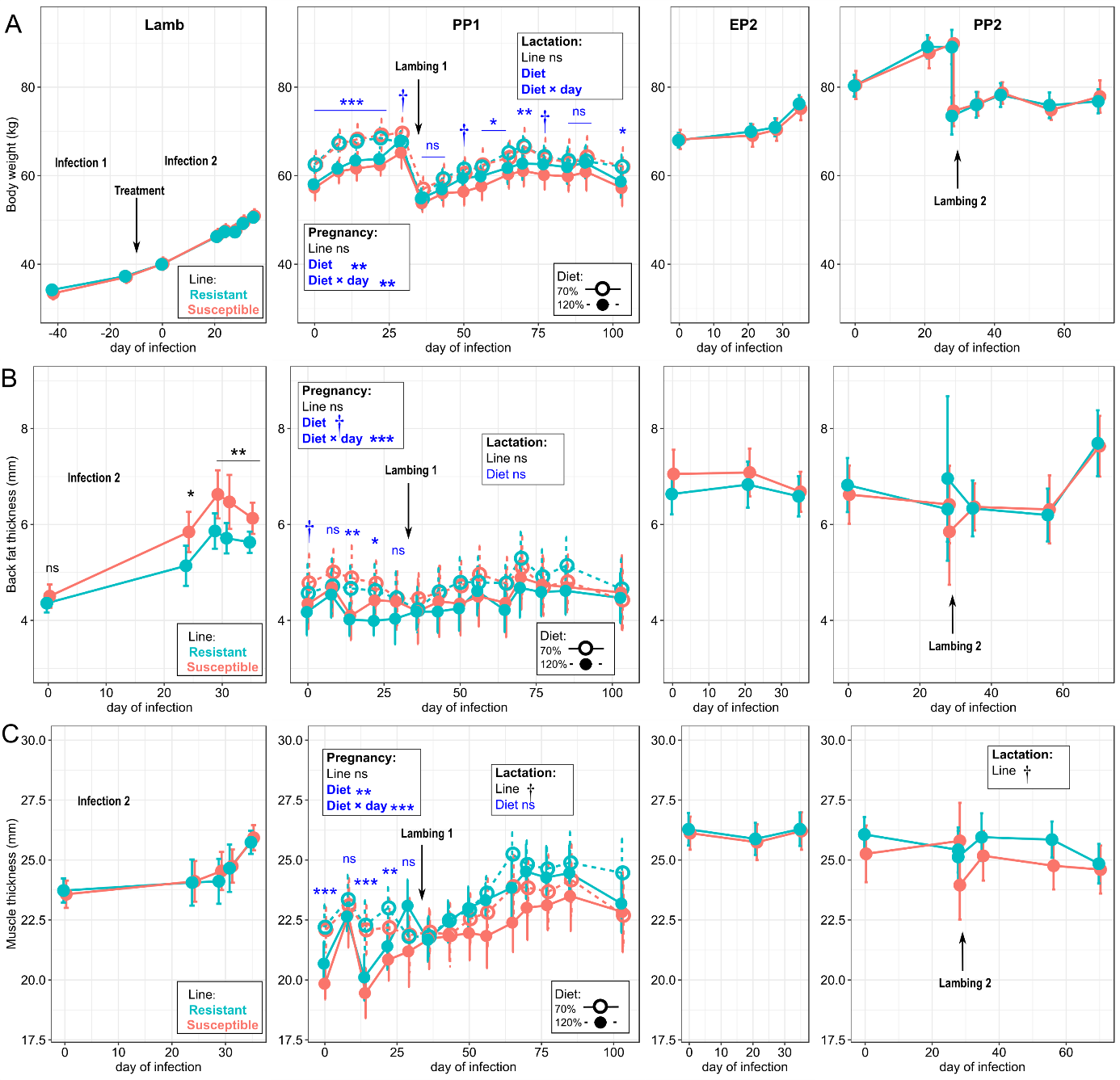


**Figure S2**: Condition traits (Body weight (A), backfat thickness (B) and muscle thickness (C)) in response to successive infections in female sheep divergently selected on resistance to *H. Contortus*. Circles are adjusted means with their error bars representing 95% confidence interval. See details about stages and infections in Figure 2. Asterisks indicate statistical differences between lines († = p < 0.1; * = p < 0.05; ** p < 0.01; *** p < 0.001).
