# Supplementary material for: The cost of host genetic resistance on body condition: Evidence from divergently selected sheep
Source: Evol Appl. 2022 Jul 12;15(9):1374–89. doi: 10.1111/eva.13442 (PMC9488686; doi:10.1111/eva.13442)
Supplement: Supplementary file 4 — Figure S4 [file EVA-15-1374-s001.docx]

**Figures S4. Changes in lamb body weight during infection at first and second peripartum**


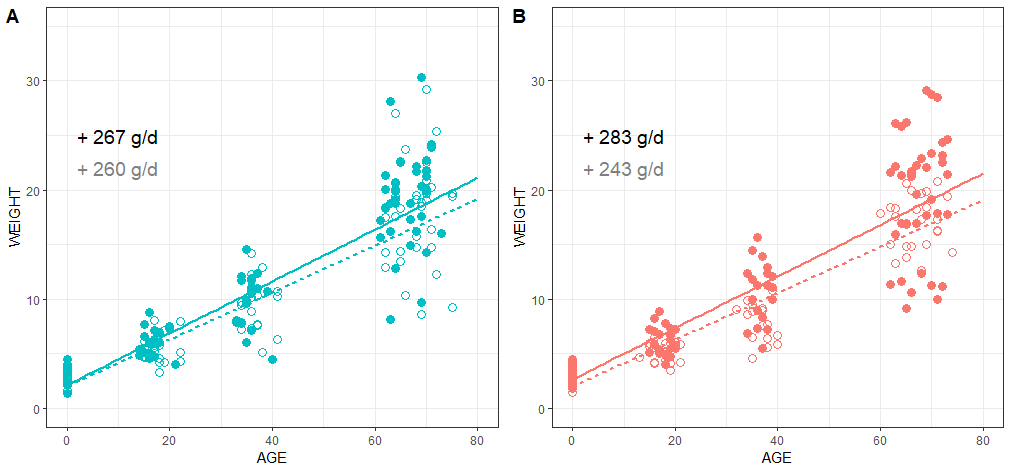


**Figure S4.1**: Change in lamb body weight with age from birth to weaning during PP1 infection in line selected for host resistance (**A**) or susceptibility (**B**). Circles indicate raw data. Lambs are reared by ewes fed a low-protein diet (empty circles) or high-protein diet (filled circles). Lines (and their slope in g/d) are predictions from linear mixed models including the interaction between age, line and diet (dashed line, grey text = low-protein diet; continuous line, dark text = high protein diet). Among those three factors, the only significant interaction was between age and diet; see details of the selected model in Table S6.


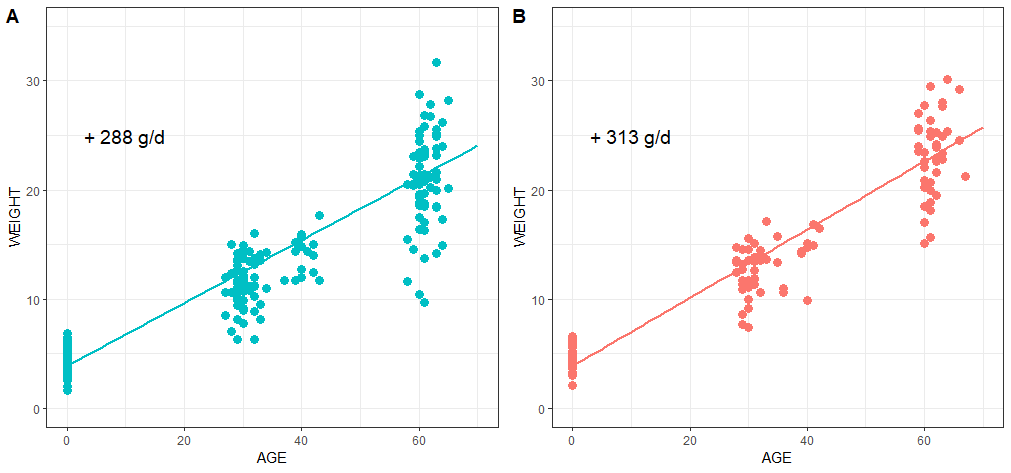


**Figure S4.2**: Change in lamb body weight with age from birth to weaning during PP2 infection in line selected for host resistance (**A**) or susceptibility (**B**). Circles indicate raw data. All lambs are reared by ewes fed a high-protein diet. Lines (and their slope in g/d) are predictions from linear mixed models including the interaction between age and line. This interaction was between age and line was significant; see details of the selected model in Table S6.
