# Supplementary material for: The cost of host genetic resistance on body condition: Evidence from divergently selected sheep
Source: Evol Appl. 2022 Jul 12;15(9):1374–89. doi: 10.1111/eva.13442 (PMC9488686; doi:10.1111/eva.13442)
Supplement: Supplementary file 6 — Appendix S1 [file EVA-15-1374-s002.docx]

### Supplementary analysis: correlations between stage-specific responses to infection

**Methods**

We measured individual covariation in parasite resistance between the different phases of infections based on multivariate modelling (as presented in Material and Methods). We considered FEC (transformed) or ΔHE at specific infection and stage (i.e. ‘Lamb’, ‘PP1-Pregnancy’, ‘PP1-Lactation’, ‘EP2’, ‘PP2-Pregnancy’, ‘PP2-Lactation’) as different traits. Random individual and residual effects were assumed to follow a multivariate normal distribution ((MVN(0, Ω_ind_) and MVN(0, Ω_e_), respectively). In addition to each trait variance within- and among-indvidual (V_e_ and V_ind_), we also estimated the covariation among-individual between traits. As, by definition, the traits were not measured simultaneously, their residual covariation was set to 0. Thus, for each infection metric (FEC and ΔHE) we modelled two 6 × 6 variance–covariance matrices (fully unstructured for Ω_ind_ and diagonal with heterogeneous variance for Ω_e_):

$$\Omega_{ind}= \left( \begin{matrix} V_{{ind}_{Lamb}} & \mathrm{COV}_{{ind}_{Lamb, PP1preg}} & \mathrm{COV}_{{ind}_{Lamb, PP1lact}} & \mathrm{COV}_{{ind}_{Lamb, EP2}} & \mathrm{COV}_{{ind}_{Lamb, PP2preg}} & \mathrm{COV}_{{ind}_{Lamb, PP2lact}} \\ \mathrm{COV}_{{ind}_{Lamb, PP1preg}} & V_{{ind}_{PP1preg}} & \mathrm{COV}_{{ind}_{PP1preg, PP1lact}} & \mathrm{COV}_{{ind}_{PP1preg, EP2}} & \mathrm{COV}_{{ind}_{PP1preg, PP2preg}} & \mathrm{COV}_{{ind}_{PP1preg, PP2lact}} \\ \mathrm{COV}_{{ind}_{Lamb, PP1lact}} & \mathrm{COV}_{{ind}_{PP1preg, PP1lact}} & V_{{ind}_{PP1lact}} & \mathrm{COV}_{{ind}_{PP1lact, EP2}} & \mathrm{COV}_{{ind}_{PPlact, PP2preg}} & \mathrm{COV}_{{ind}_{PP1lact, PP2lact}} \\ \mathrm{COV}_{{ind}_{Lamb, EP2}} & \mathrm{COV}_{{ind}_{PP1preg, EP2}} & \mathrm{COV}_{{ind}_{PP1lact, EP2}} & V_{{ind}_{EP2}} & \mathrm{COV}_{{ind}_{EP2, PP2preg}} & \mathrm{COV}_{{ind}_{EP2, PP2lact}} \\ \mathrm{COV}_{{ind}_{Lamb, PP2preg}} & \mathrm{COV}_{{ind}_{PP1preg, PP2preg}} & \mathrm{COV}_{{ind}_{PPlact, PP2preg}} & \mathrm{COV}_{{ind}_{EP2, PP2preg}} & V_{{ind}_{PP2preg}} & \mathrm{COV}_{{ind}_{PP2preg, PP2lact}} \\ \mathrm{COV}_{{ind}_{Lamb, PP2lact}} & \mathrm{COV}_{{ind}_{PP1preg, PP2lact}} & \mathrm{COV}_{{ind}_{PP1lact, PP2lact}} & \mathrm{COV}_{{ind}_{EP2, PP2lact}} & \mathrm{COV}_{{ind}_{PP2preg, PP2lact}} & V_{{ind}_{PP2lact}} \end{matrix} \right)$$

and

$$\Omega_{e}= \left( \begin{matrix} V_{e_{Lamb}} & 0 & 0 & 0 & 0 & 0 \\ 0 & V_{e_{PP1preg}} & 0 & 0 & 0 & 0 \\ 0 & 0 & V_{e_{PP1lact}} & 0 & 0 & 0 \\ 0 & 0 & 0 & V_{e_{EP2}} & 0 & 0 \\ 0 & 0 & 0 & 0 & V_{e_{PP2preg}} & 0 \\ 0 & 0 & 0 & 0 & 0 & V_{e_{PP2lact}} \end{matrix} \right)$$

The fixed effects were determined from univariate modelling (Table S1-S4). However, we considered two cases: in one case ‘Line’ and its potential interaction with other factors was excluded from the fixed effects, whereas those effects were included in the other case (i.e. part of the variance due to difference between lines was accounted for in the fixed structure). The corresponding among-individual correlations were called r_total_ and r_marginal_, respectively. If infection traits were mostly correlated between lines, then r_total_ would be generally higher than r_marginal_. A significant r_marginal_ would suggest that stage-specific responses to infection are also correlated within-individual (assuming a unique correlation for both lines).

We tested the significance of a particular correlation r_ind_ using likelihood ratio test (likelihood comparison of the full model estimating all covariance terms to the model where the particular r_ind_ is constrained to zero).

**Results**

Parasite resistance expressed during the three adult stages (from PP1 to PP2) was strongly positively correlated to resistance expressed in the lamb stage and on which selection was based (r_total_ > 0.5, except for pregnancy 1 where too few data on FEC were available, see first column in Table i; details in Table S8). Those correlations were close to those observed between FEC responses in the adult stages. Moreover, they persisted even when individual variation due to the line effect was accounted for (correlations r_marginal_ above the diagonal, Table i). Overall, adult parasite resistance was thus consistent with the selected resistance in lambs.

For ΔHE, r_total_ were less consistent between the lamb stage and the adult stages, and were not detected once the line effect has been accounted for (Table i).

**Table i**: Phenotypic correlations between responses to infection during successive stages in female sheep divergently selected for resistance to *H. contortus*. Correlations r_total_ are below the diagonal whereas correlations r_marginal_ are above the diagonal.

| **Trait** | | **FEC^b^** | | | | | | **ΔHE** | | | | | |
| --- | --- | --- | --- | --- | --- | --- | --- | --- | --- | --- | --- | --- | --- |
| **Phase at infection ^a^** | | Lamb | PP1 | | EP2 | PP2 | | Lamb | PP1 | | EP2 | PP2 | |
|  |  |  | Preg. | Lact. |  | Preg. | Lact. |  | Preg. | Lact. |  | Preg. | Lact. |
| **Lamb** | |  | 0.45  * | 0.39  * | 0.48  *** | 0.43  * | - |  | - | - | - | - | - |
| **PP 1** | Preg. | - |  | - | - | - | - | - |  | 0.97  *** | - | - | - |
|  | Lact. | 0.55  *** | - |  | 0.71  *** | 0.50  * | 0.53  * | - | 0.96  *** |  | - | - | - |
| **EP 2** | | 0.65  *** | - | 0.79  *** |  | 0.46  * | 0.67  *** | 0.41  ** | - | 0.33  * |  | - | - |
| **PP2** | Preg. | 0.58  ** | - | 0.61  ** | 0.58  *** |  | 0.86  *** | - | 0.59  * | 0.60  * | - |  | 0.79  *** |
|  | Lact. | 0.52  ** | - | 0.65  ** | 0.79  *** | 0.92  *** |  | - | 0.39  † | 0.52  ** | 0.39  ** | 0.83  *** |  |

^a ‘^Lamb’: single-dose infection at 4-5 months of age; ‘PP1’ or ‘PP2’: peripartum infection at 1 year of age (trickle) or at 2 years of age (single-dose); late pregnancy (Preg) and lactation (Lact) analysed separately; ‘EP2’: single-dose infection during early pregnancy 2

^b^ transformed as log(FEC + 1) for Lamb, PP1_Preg_ and PP2_Preg_, and as $\sqrt{FEC}$ for PP1_Lact_, EP, and PP2_Lact_
